# Design of bacterial DNT sensors based on computational models

**DOI:** 10.1093/nar/gkaf1482

**Published:** 2026-01-08

**Authors:** Shir Bahiri Elitzur, Etai Shpigel, Itay Katzir, Uri Alon, Shimshon Belkin, Tamir Tuller

**Affiliations:** Department of Biomedical Engineering, Tel Aviv University, Chaim Levanon St 55, Tel Aviv-Yafo 6997801, Israel; Department of Plant and Environmental Sciences, Institute of Life Sciences, The Hebrew University of Jerusalem, Edmond J. Safra Campus, Jerusalem 9190401, Israel; Department of Molecular Cell Biology, Weizmann Institute of Science, Herzl St 234, Rehovot 7610001, Israel; Department of Molecular Cell Biology, Weizmann Institute of Science, Herzl St 234, Rehovot 7610001, Israel; Department of Plant and Environmental Sciences, Institute of Life Sciences, The Hebrew University of Jerusalem, Edmond J. Safra Campus, Jerusalem 9190401, Israel; Department of Biomedical Engineering, Tel Aviv University, Chaim Levanon St 55, Tel Aviv-Yafo 6997801, Israel; Sagol School of Neuroscience, Tel Aviv University, Chaim Levanon St 55, Tel Aviv-Yafo 6997801, Israel

## Abstract

Detecting explosive compounds, such as 2,4,6-trinitrotoluene and its volatile byproduct 2,4-dinitrotoluene (DNT), is paramount for public health and environmental safety. In this study, we present the successful application of diverse computational and data analysis models toward developing a bacterial biosensor engineered to detect DNT with high sensitivity and specificity. The *Escherichia coli-*based biosensor harbors a plasmid-based fusion of a gene promoter, acting as the sensing element, to a microbial bioluminescence gene cassette as the reporter. By analyzing endogenous and heterologous promoter data under conditions of DNT exposure, a total of 367 novel variants were generated. The biosensors engineered with these modifications demonstrated a remarkable amplification of up to four-fold change in signal intensity upon exposure to 2,4-dinitrotoluene, compared to non-modified biosensors, accompanied by a decrease in the detection threshold and a shortening of the response times. Our analysis suggests that the sequence features with the highest contribution to biosensor performance are DNA folding patterns and nucleotide motifs associated with DNT sensing. These computational insights guided the rational design of the biosensor, leading to significantly improved DNT detection capabilities compared to the original biosensor strain. Our results demonstrate the effectiveness of integrating computational modeling with synthetic biology techniques to develop advanced biosensors tailored for environmental monitoring applications. A similar approach may be applied to a wide array of ecological, industrial, and medical sensing endeavors.

## Graphical Abstract

**Graphical Abstract:**
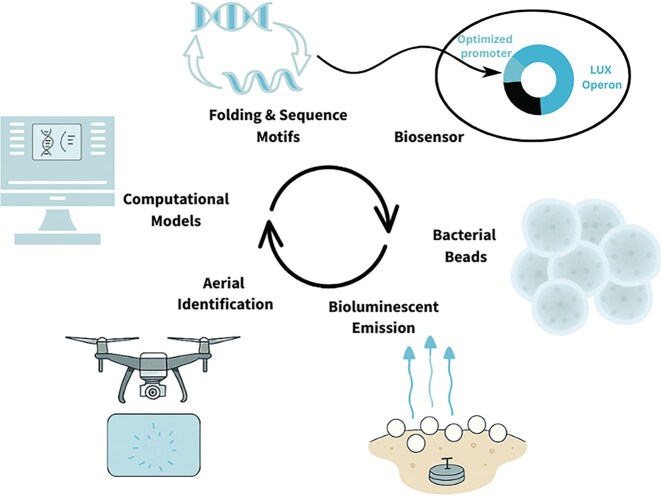

## Introduction

The detection of unexploded antipersonnel mines is a pressing global concern, impacting millions worldwide. Despite efforts by developed nations and humanitarian organizations to clear these mines, they continue to pose a significant threat, resulting in an estimated 15 000–20 000 casualties annually [1]. Beyond the humanitarian aspects, the presence of landmines disrupts various facets of life, ranging from agricultural activities to commercial endeavors. Despite ongoing clearance initiatives, the sheer magnitude of the task suggests that eliminating all current existing mines may require as long as 450–500 years at the current rate [1, 2].

Presently, the predominant technologies for landmine detection mirror those employed during World War II, typically involving the use of metal detectors [3]. Not only do these methods yield high false-positive rates, but they also fail to detect non-metallic munitions, which constitute most modern landmines. Moreover, the need for the presence of personnel on the actual minefield poses a significant risk of injury or death [4]. At present, there is no viable technology for the standoff detection of buried explosives.

In response to these challenges, alternative methods have been pursued, aiming to significantly reduce the false-positive rate while maintaining a high probability of detection, thereby enhancing efficiency and minimizing the risk of harm. Such approaches encompass electromagnetic, acoustic, and explosive-vapor sensing technologies, among others [5, 6].

Recently, the use of genetically engineered bacteria has been reported to have explosive detection capabilities. Furthermore, this *Escherichia coli*-based biosensor is activated by exposure to 2,4,6-trinitrotoluene (TNT) and 2,4-dinitrotoluene (DNT), with a modified version of the *yqjF* gene promoter (C55) [7, 8] as the sensing element, coupled with bioluminescent proteins encoded by a bacterial (*Aliivibrio fischeri*) *luxCDABE* gene cassette [9] as the reporter element. However, despite these advancements, the sensor’s performance can be further improved. However, the sensitivity, response time, and detection threshold of the sensor remain suboptimal, highlighting the need for further optimization to enable reliable real-world deployment. For example, performance could be improved by performing additional design–build–test iterations, optimizing codon usage to enhance gene expression, applying metabolic engineering to redirect cellular resources toward reporter output, and leveraging AI-guided design tools to explore a broader and more efficient sequence space.

This approach exemplifies a broader class of whole-cell biosensors, which leverage the natural responsiveness of microorganisms to detect and report environmental changes through genetically encoded outputs.

A biosensor detects and records physiological or biochemical changes, relying on the specificity of its biological material. Key features include specificity, sensitivity, and real-time analysis. Microorganisms, particularly bacteria, are advantageous as biosensors due to their adaptability, their broad-spectrum molecular/biochemical responses to toxic threats, their ability to metabolize diverse compounds, and ease of genetic manipulation. The term “whole-cell biosensor,” as used in this article, refers to live cells that respond to the presence of a target compound in their environment by generating a dose-dependent, quantifiable physical signal. Such biosensors have been demonstrated to track natural signaling pathways by controlling gene expression, allowing precise monitoring of cellular responses [10–20].

The aforementioned studies emphasize the effectiveness of bacterial biosensing, as evidenced by compelling proofs of concept. Specifically, the use of genetically engineered microbial biosensors presents a promising biological approach for the remote detection of landmines [21]. The concept revolves around the use of bacteria to generate an optical signal (reporter gene expression) in the presence of explosive vapors, enabling the detection of explosive devices from a safe distance [22, 23]. Over the years, several strains of explosives-sensing bacteria have been reported, culminating in recent advancements such as the remote detection of buried antipersonnel landmines using an *E. coli-*based biosensor [24–30].

Previous research has demonstrated how computational models can be used to analyze and solve complex problems in synthetic biology. For instance, one important aspect is predicting how molecules such as proteins and nucleic acids interact from a thermodynamic perspective [31]; kinetic models are also commonly used to control gene expression over time, allowing for the design of complex behaviors, such as oscillations [32]. Another important area where such models can have an impact is the design of regulatory circuits, which are crucial for controlling cellular processes [33–35]. Computational models also help to predict changes in microbial metabolism to improve bioprocess productivity [36–39]. When we insert foreign genes into an organism, it is vital to construct stable genetic constructs that remain functional for extended periods [40]. Computational models also help us address other critical aspects of synthetic biology [41–43].

Another approach uses endogenous gene expression data [44–47] due to their reliability. However, endogenous transcripts are influenced by various forms of evolutionary selection and possess diverse characteristics, such as amino acid content and promoters, making it challenging to establish causality solely through their analysis. To address these challenges, an analysis of synthetic biology libraries may be employed. For instance, a study conducted to investigate how protein abundance is encoded in different transcript regions enabled researchers to identify new causal relationships between features in previously unexplored regions of transcripts and natural gene expression patterns [48]. Another study demonstrated how synthetic libraries can predict the fine-tuning of synthetic gene expression systems in yeast by incorporating various predictive intron features, such as transcript folding and sequence motifs [49]. Researchers have also created synthetic promoter libraries to enhance the expression of desired proteins [50]. While numerous papers emphasize the effectiveness of utilizing synthetic biology libraries in research [51, 52], to the best of our knowledge, no such approach has been used in the field of biosensors.

The present study’s objective was to enhance the explosives’ detection capabilities of an existing microbial biosensor strain to improve its amenability to field requirements. This study combines computational modeling of gene expression with synthetic biology techniques to enable the rational design and development of a whole-cell biosensor [53]. While our primary focus lies in detecting buried landmines as a prototype system, the adaptable nature of this approach renders it applicable to a broad spectrum of environmental, industrial, and medical sensing scenarios.

## Methods

### Chemicals and reagents

DNT (2,4-dinitrotoluene) was purchased from Sigma–Aldrich (cat. 101 397). A working stock of 10 g/l in ethanol was prepared and kept at room temperature.

### Plasmids and strains

**Figure 1.**
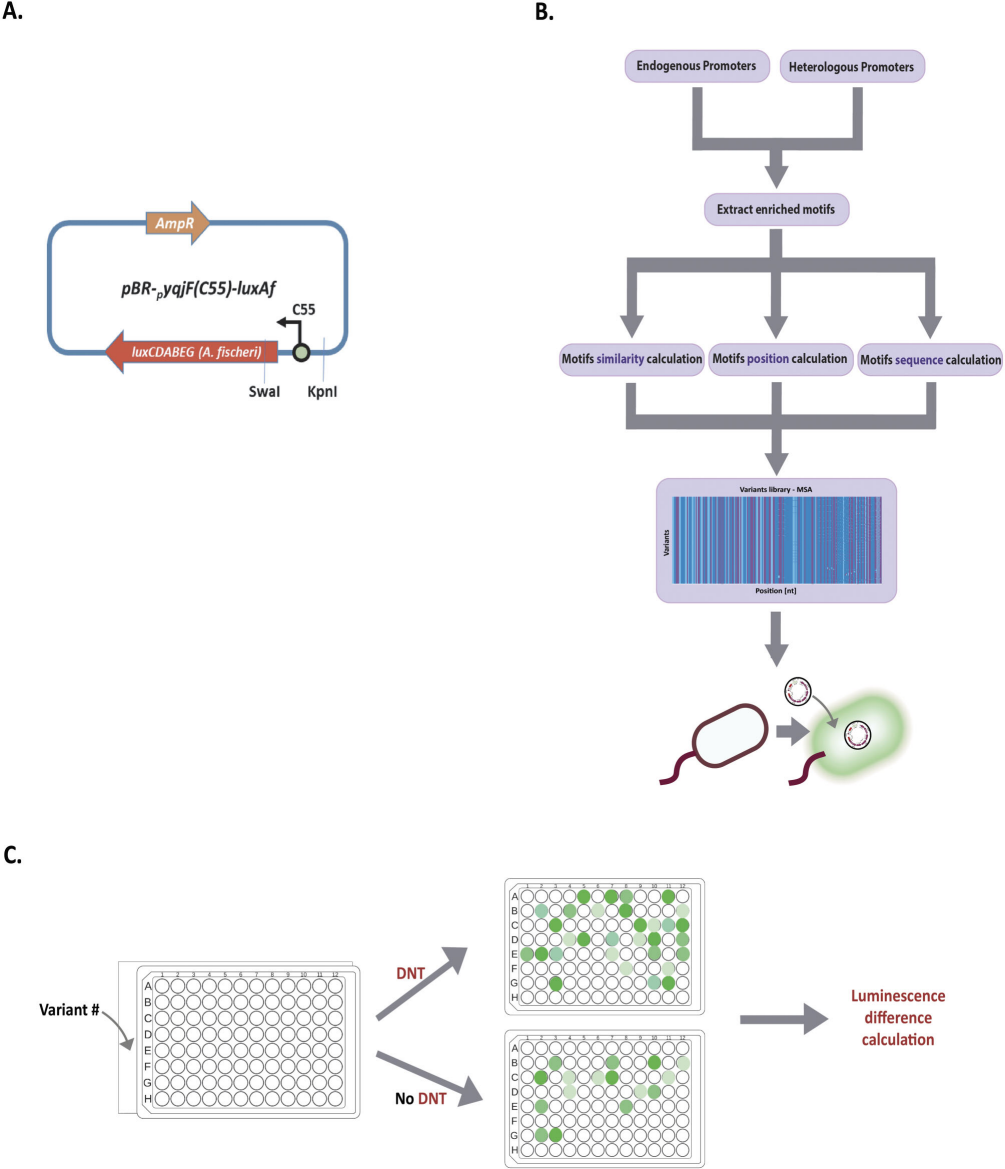
(A) Scheme of the plasmid used in this study (pBR-C55-luxAf) with the KpnI and SwaI restriction sites. The yqjF (C55 version) gene promoter optimized during the research is marked. (B) An illustration of the computational pipeline employed. (C) Experiment illustration.

The previously described [52] plasmid pBR-C55-luxAf, harboring the *Aliivibrio fischeri luxCDABE* gene cassette downstream of the *yqjF* gene promoter (version C55), served as the chassis for testing the different *yqjF* variants^69^ (Fig. 1A). *Escherichia coli* strain BW25113 [54] served as the host in all experiments.

### The analyzed data libraries

To enhance the biosensor’s detection capability and sensitivity, two datasets were used: the endogenous genes dataset and the heterologous genes dataset. The goal was to address the limitations of each, specifically resolving causality issues in the endogenous dataset and improving specificity in the heterologous dataset.

### Endogenous promoters library data

We utilized RNA-seq data from *E. coli* genes, both with and without exposure to DNT (5 mg/l) [55], to identify endogenous genes exhibiting either over-expression or under-expression upon DNT exposure. For each gene, we quantified the expression disparity and selected the promoters (each with a length of 400) corresponding to the over-expressed genes and a subset representing the under-expressed genes (10%). This dataset is denoted as Dataset A.

### Heterologous promoters library data

The synthetic library, detailed in Supplementary Table S2, is based on the *yqjF* gene promoter fused with GFP, and includes single mutations across 147 sites, as well as various mutation pairs, triplets, and higher-order combinations, totaling approximately 100 000 sequences. To assess promoter activity, flow-seq method [56] was employed, and sorted the library into 16 logarithmic bins based on GFP fluorescence using Fluorescence-Activated Cell Sorting, as shown in Supplementary Fig. S1. This sorting was performed twice, once with DNT (200 μg/ml) and once without.

To identify motifs potentially influencing expression in response to DNT exposure, we categorized the promoters into over-expressed and under-expressed groups, each comprising the top 10% based on the weighted average fluorescence of individual promoters:


(1) \begin{eqnarray*}
{{F}_i} = \frac{{\mathop \sum \nolimits_j {{b}_i}{{n}_{ij}}}}{{{{N}_i}}}.
\end{eqnarray*}

Where ${{b}_i}$ is the average fluorescence in bin *i, nij* is the number of appearances of variant *j* in bin *i*, and ${{N}_i}$is the total count of variant *i*.

We refer to this data set as Dataset B.

### Sensing element (C55 promoter) optimization

To optimize the whole-cell biosensor and improve its DNT sensing capability, we focused on enhancing the C55 promoter within the plasmid, based on the *yqjF* gene promoter, as shown in Fig. 1A. The optimization was tailored for each dataset and involved the following steps:

**Step 1 – Sequence motif extraction**

We used a differential enrichment algorithm to discover significant motifs by comparing target and reference sets. Searches were conducted with the following configurations:

1. Over-expressed promoters as the target set, and all promoters as the reference.
2. Over-expressed promoters as the target set, and under-expressed promoters as the reference.
3. Under-expressed promoters as the target set, and all promoters as the reference.
4. Under-expressed promoters as the target set, and over-expressed promoters as the reference.

This process was applied separately to Dataset A and Dataset B, resulting in 159 significant motifs for Dataset A, and 596 for Dataset B.

**Step 2 – Find similar motifs**

To streamline the motif pool and ascertain consistency across motifs derived from both datasets (A and B), we aimed to identify similarities between motifs. Each motif was represented by a position-specific scoring matrix (PSSM). To facilitate comparison between motifs, a distance metric was employed:


(2) \begin{eqnarray*}
\text{Similarity}\ \text{score} &=& \frac{1}{{\text{motif}\ \text{length}}}\mathop \sum \limits_i^{\text{motif}\ \text{length}} \\ &-& \frac{{\text{Observed}_i - \text{Expect}_i}}{{\text{Expect}_{i}}},
\end{eqnarray*}

(3) \begin{eqnarray*}
\text{Observed}{_i} = \mathop \sum \limits_j^{A,C,G,T} - {{\left( {\textit{freq}_1^{i,j} - \textit{freq}_2^{i,j}} \right)}^2},
\end{eqnarray*}

(4) \begin{eqnarray*}
\text{Expected}_{i} = \mathop \sum \limits_j^{A,C,G,T} \mathop \sum \limits_k^{A,C,G,T} \frac{{ - {{{\left( {\textit{freq}_1^{i,j} - \textit{freq}_2^{i,j}} \right)}}^2}}}{4}.
\end{eqnarray*}

To compare PSSMs, we standardized the number of rows and columns by applying equal padding (0.25) to motifs of varying lengths. Padding was uniformly added along the edges in all configurations, as shown in Supplementary Fig. S2A, with the final score being the maximum similarity score.

To set a meaningful threshold, we used an empirical *P*-value test. We permuted the rows (nt) of the PSSMs 100 times and computed random similarity scores, as illustrated in Supplementary Fig. S2B. A score was considered significant if it was ≤0.05.


(5) \begin{eqnarray*}
{{P}_{\text{value}}} = \frac{{\text{number}\ \text{of}\ \text{times}\left( {\text{real}\ \text{score} \le \text{random}\ \text{score}} \right)}}{{100}}.
\end{eqnarray*}

For each motif in Dataset A, we compared it with all motifs in Dataset B and selected the one with the highest similarity score. Motifs with the highest similarity scores illustrated in Supplementary Fig. S2C. This process identified 73 motifs with significant similarity and relevance.

**Step 3 – Find significant motif positions**

The discovered motif is linked to target and reference sequences and a PSSM matrix. To find the optimal insertion site within the C55 promoter, we maximize or minimize the PSSM score based on whether the motif is from over- or under-expressed promoters.

The process for identifying the most influential insertion site involves:

1. Selecting a motif.
2. Choosing a relevant promoter for the motif.
3. Calculate the PSSM score at all positions within the promoter using a sliding window.
4. Generating 100 permutations of the promoter by shuffling nucleotides while preserving nucleotide distribution and GC content.
5. Calculate the PSSM score for the motif at all positions in each permuted promoter.
6. Determining the empirical *P*-value for each position to identify significant sites.

This process is repeated for all motifs and their respective promoters, as shown in Supplementary Fig. S3A.

It is noteworthy that each motif may have multiple significant positions across different promoters. Thus, our objective was to identify the most prevalent position for each motif. To achieve this, the following additional steps were undertaken:

1. Calculate the frequency of occurrence (x_i) for each significant position across all relevant promoters.
2. Expand each position into a window spanning ± 4 nucleotides to provide greater flexibility.

Step 3: Calculate the sum of appearances for each window using Equation (6):
  (6) \begin{eqnarray*}
{{X}_j} = \mathop \sum \limits_i {{x}_i}.
\end{eqnarray*}
Step 4: Compute the percentage of appearances for each window using Equation (7):
  (7) \begin{eqnarray*}
\text{window}{_j} = \frac{{{{X}_j}}}{{\mathop \sum \nolimits_j {{x}_j}}} \cdot 100.
\end{eqnarray*}
Step 5: Select thewindow with the maximum percentage of appearances for each motif (max($\mathrm{ window}{_j}$)).

Motifs were selected if they appeared in windows with a percentage greater than 6.5%, ensuring a sufficient number of motifs. Due to lower percentages in Dataset A, motifs from this dataset were matched with similar motifs from Dataset B (see Supplementary Fig. S3B and C).

For motifs from both datasets, significant positions were those that were identical or within ± 20 nt.

The final step was to determine the optimal insertion position for each motif by calculating and comparing PSSM scores at relevant positions within the C55 promoter and the corresponding promoters from both datasets, as outlined in Equations (8) and (9). The goal was to insert motifs from over-expressed promoters, and remove those from under-expressed ones. Promoter lengths varied, so positions were aligned to the START codon.

For motifs from over-expressed promoters, the selected positions had to meet the following conditions:


(8) \begin{eqnarray*}
\mathrm{ max}(\mathrm{ score}{_{C5 - 5}})< {\mathrm{max}}(\overline {\text{Score}{_{\text{promoters}}})}.
\end{eqnarray*}

Where $\mathrm{ score}{_{C5 - 5}}\ $is a vector of PSSM’s scores of a motif in all its significant position in the C55 promoter, $\overline {\text{Score}{_{\text{promoters}}}} $ is the average score of PSSM’s scores of a motif in all its significant position and all relevant promoters.

For motifs from under-expressed promoters, the selected positions met the following conditions:


(9) \begin{eqnarray*}
\mathrm{ max}(\mathrm{ score}{_{C5 - 5}}) > {\mathrm{max}}(\overline {\text{Score}{_{\text{promoters}}})}.
\end{eqnarray*}

Where $\mathrm{ score}{_{C5 - 5}}\ $is a vector of PSSM’s scores of a motif in all its significant position in the C55 promoter, $\overline {\text{Score}{_{\text{promoters}}}} $ is the average score of PSSM’s scores of a motif in all its significant position and all relevant promoters.

After this process, we identified 13 motifs from Dataset A and 15 motifs from Dataset B.

**Step 4 – Find the final motif’s sequence**

Since determining the consensus sequence of a motif from a PSSM matrix is not straightforward, we developed an algorithm to maximize the PSSM score of a motif at its designated position. The goal is to optimize the motif’s impact by maximizing its score, as outlined in Supplementary Fig. S4A.

Algorithm steps:

1. *Initialization*: Compute the initial PSSM score of the motif at its position in the promoter.
2. *Iteration*:
  - Generate a pool of sequences with single mutations.
  - For each position within the motif, substitute the nucleotide with each of the other three possible nucleotides.
  - Calculate the PSSM score for each mutated sequence.
  - Select the sequence with the highest score change compared to the previous iteration.
  - Repeat the process without altering the position changed in the last iteration.
3. *Termination*: The algorithm stops when no further improvements are observed, and the final sequence is chosen as the optimal motif sequence.

Supplementary Fig. S4B illustrates an iteration of the algorithm, showing score improvements and nucleotide changes.

**Step 5 – Insert motif into the promoter**

This phase involves constructing a library of variants. Initially, positions that are constrained by factors such as restriction sites are identified and marked, precluding any alterations. Subsequently, each motif is individually inserted into the plasmid. Finally, motifs from both datasets are concurrently incorporated into the same variant using a Cartesian product approach, with the condition that they do not overlap.

### Construction of the *yqjF* variant library

All variant *yqjF* promoter sequences were synthesized and cloned by Twist Bioscience (San Francisco, CA, USA) and were obtained pre-cloned, at KpnI/SwaI sites, in a pBR-X-luxAf backbone (*Aliivibrio fischeri luxCDABE*). Overall, 397 clones were ordered, 38 of which failed Twist Bioscience QC before synthesis. Hence, 367 clones were successfully synthesized and tested for their reaction to DNT.

### Bioluminescence assays

Each clone was transformed separately into *E. coli* DH5α and grown overnight (37°C, 200 rpm rotary shaking) in 96-well plates, each well containing 150 µl lysogeny broth (LB) with 100 µg/ml ampicillin. Overnight culture aliquots were transferred into individual wells of duplicate 96-well plates, each well containing 50 µl of the same medium. Following 3 h growth under the same conditions, 50 µl of DNT dissolved in LB (final concentration 5 mg/l) was added to one plate, and the same volume of DNT-free LB was added to the other. Both plates were incubated at 25°C for 10 h, in the course of which bioluminescence and optical density at 600 nm were measured every hour using a Microlab Star robot (HAMILTON, Switzerland) and a Synergy HT, BioTek plate reader. The following values were recorded for each clone: maximum luminescence intensity, net luminescence (after subtraction of the control luminescence), and response ratio (luminescence divided by the control luminescence). The five best-performing clones were selected based on the following criteria: having both the highest net luminescence and the highest ratio along with faster signal development.

The response of the selected clones to DNT was analyzed by exposing them to a DNT concentration series and measuring the ensuing luminescence as a function of time.

Grown overnight at 37°C with vigorous agitation in LB supplemented as before. The culture was diluted x1/100 in LB without antibiotics and regrown under the same conditions to an OD_600_ = 0.3. Bacterial aliquots (50 µl) were transferred to an opaque white 96-well plate (Greiner Bio-One), already containing 50 µl of DNT at various concentrations in 4% ethanol (2% final concentration). Bioluminescence was measured every 15 min at ambient temperature, using a microplate reader (either a TECAN Infinite® 200 PRO, Männedorf, Switzerland, Synergy HT, BioTek or a Wallac VICTOR2, Turku, Finland). All experiments were conducted with internal duplicates, were repeated at least three times, and pBR-C55-luxAf served as the control strain.

Luminescence data are presented either as the instrument’s raw arbitrary relative light units (RLU) or as the net signal in the presence minus the absence of the inducer (ΔRLU). The detection sensitivity of the biosensors to DNT is presented as the EC_200_ value, representing the DNT concentration at which luminescence in the presence of DNT is twice that in its absence [57, 58].

### Bioluminescence assay on sand

For testing the response to DNT of the bacterial bioreporters on a solid matrix, they were immobilized in 3–4 mm diameter alginate beads (2.5% alginate, 0.5% polyacrylic acid), as previously described [52], and kept at 4°C until use. To evaluate the bacterial response to DNT on sand, 11-gram aliquots of beach sand were dispensed into the wells of a six-well microtiter plate (Greiner Bio-One). Different DNT concentrations in 20 μl of 100% ethanol were added to the center of each well, and the ethanol was allowed to evaporate for 1 h at room temperature. Before the experiment, the encapsulated bacteria were removed from refrigeration and incubated in LB for 4 h at 37°C with shaking (200 rpm). The beads were then drained of medium and placed in a single layer over the sand surface of the microtiter plate wells. Plates were incubated for 10 h at room temperature in a dark chamber; images of the bioluminescent response were acquired every 20 min using a Sony α7SII camera equipped with a 28 mm lens (5 s exposure, f = 2, ISO = 5000).

### Computational analysis of the library

**Figure 2.**
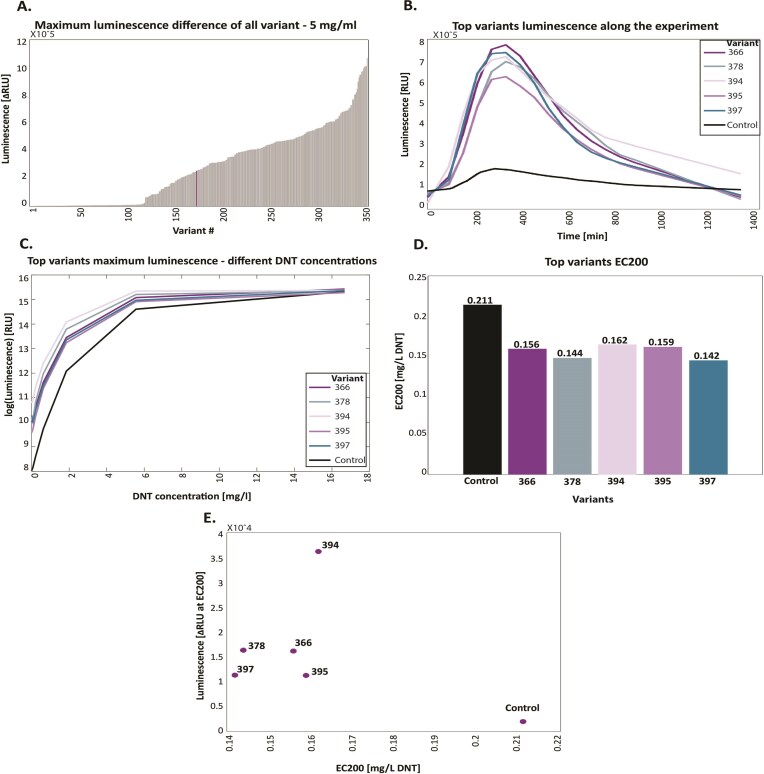
(A) Library scan for response to DNT at 25°C. All variants are sorted by maximum luminescence difference net value. The purple line represents the unmodified control variant (average of three repeats). (B) Luminescence development over time of the top five variants and the unmodified control variant in the presence of 5 mg/l DNT (extrapolation of the experiment data to match all the time points of all experiments; more explanations in the “Methods” section). Luminescence data are provided in the plate reader’s arbitrary relative luminescence units (RLU). (C) Maximum luminescence over a 5-h exposure as a function of DNT concentration. (D) Detection threshold, presented as the EC200 value (DNT concentration eliciting a two-fold increase in bioluminescence compared to the uninduced control). (E) Net signal intensity (ΔRLU, luminescence in the presence of DNT minus that in its absence) at a DNT concentration equal to EC200, plotted against the EC200 values.

To analyze the variants’ behavior, we compared luminescence data from different plates, requiring interpolation and extrapolation to standardize measurements across consistent time points. As shown in Fig. 2A, the variants displayed varied behaviors, prompting an investigation into their sequences to understand the luminescence results.

### Extracted features

We extracted 6711 sequence-related features from the promoters of the variants, including the following categories (can be seen in Supplementary Table S3):

*Folding energy*: the folding energy of the variants’ promoters was calculated using the MATLAB function “rnafold.”
*Folding in windows*: calculation of the folding energy for every 40 nucleotides with a sliding window of 1 (the window’s position is determined by the first nucleotide in the window).
*Average folding*: the average folding value across all windows.
*Total folding*: the folding energy of the entire variant.

1. *Mutations*: each promoter was compared to the control promoter to identify mutations.
  - *Existence of mutation*: a binary feature indicating the presence (1) or absence (0) of a mutation at a specific position.
  - Amount of mutation: the total number of mutations in a variant.
2. Chimera ARS (average repetitive substring) index [59]: this index is based on the propensity of coding regions to include long substrings that appear in other coding sequences, assumed to be regulatory regions. We calculated the average repetitive substring in our variants using a reference set (the top 20% of over-expressed *E. coli* promoters).
3. Sequence motifs:

PSSM scores [60]: maximum PSSM scores of the inserted motifs.
New motif extraction: extraction of new motifs as described in the Sequence Motifs Extraction section.
Motifs from SwissRegulon [61]: downloaded from the SwissRegulon *E. coli* database, which contains genome-wide annotations of regulatory motifs, promoters, and transcription factor binding sites (TFBSs). This set includes 37 motifs.

1. Promoter Strength [62]: calculated using an energy matrix [KBT]. The matrix covers base pairs [−41:−1], where 1 denotes the transcription start site. Each row corresponds to a nucleotide, and each column corresponds to a position.

Strength by position: promoter strength is calculated for specific positions.
Average promoter strength: overall promoter strength across the entire sequence.

1. *Nucleotide count features*: based on nucleotide counts from [63], as detailed in Supplementary Table S3 in the Supplementary section.
2. *Ribosome binding site strength*: calculated by a high-resolution computational model that predicts ribosomal RNA–Messenger RNA interactions’ strength in a sliding window with the size of six nt along the promoter [64].

This detailed extraction of sequence-related features enabled a comprehensive analysis of the variants' promoters, facilitating the understanding of their luminescence behavior.

To streamline our feature set:

1. *Folding in windows*: we calculated the Spearman correlation between folding features and retained only one representative feature for those with a correlation above 0.99.
2. *Mutations*: we averaged mutation profiles at similar positions to reduce redundancy.

These steps improved the efficiency of our analyses.

### Prediction variables

We used *K*-means clustering with correlation as the distance metric to group variants based on luminescence differences over time. After testing various *K* values, we selected *K* = 2 based on cluster evaluation metrics (Davies–Bouldin index [65] and Silhouette [66] score). As can be seen in Supplementary Fig. S5, for *K* = 2, the average luminescence differences pattern is different, and from that we chose to predict four variables: maximum luminescence difference, average luminescence difference, maximum slope value, and time to maximum luminescence difference.

### Predictor algorithm

To predict the selected variables, we employed three regression models:

1. Linear regression:

The data was split into training (60%), testing (20%), and validation (20%) sets, with each cross-validation iteration using a different random split.
Features were added using forward selection to maximize the correlation on the test set.
The process was repeated for 10 cross-validation iterations.

1. Lasso regression [67]:

The data were divided into training (80%) and validation (20%) sets.
The process was repeated for 10 cross-validation iterations.

The “lassocv” (https://scikit-learn.org/stable/modules/generated/sklearn.linear_model.LassoCV.html), function in Python was used to optimize the Lasso algorithm’s parameters, iteratively fitting along a regularization path.

1. XGBoost (eXtreme Gradient Boosting) [68]:
  - The data were split into training (60%), testing (20%), and validation (20%) sets, with each cross-validation iteration using a different random split.
  - Features were added using forward selection to maximize the correlation on the test set.
  - The process was repeated for 10 cross-validation iterations.
  - Hyperparameters were optimized using Optuna [69], which automates the search for optimal configurations through Bayesian optimization. Each trial used a different set of hyperparameters, evaluating the R-squared score on the validation set. The best hyperparameter set for our data and model was selected after adjusting the range and re-running Optuna (details provided in the supplementary section Supplementary Fig. S6).

This approach ensured that the most effective predictive model and feature set were selected, enhancing the accuracy and robustness of our predictions.

### Classification algorithm

As can be seen in Fig. 2B there is a division between the maximum luminescence difference of the variants. To examine the difference, we classified the variant into three groups according to two thresholds (relative to the control variant, while having balanced data in each label group). We used two classification models:

1. SVM (support vector machine) [70]:
  - Radial basis function (RBF) kernel.
  - The data were divided into training (80%) and validation (20%) sets with each cross-validation iteration using a different random split.
  - Features were added using forward selection to maximize the accuracy of the test set.
2. XGBoost:
  - The data were split into training (80%), and testing (20%) sets, with each cross-validation iteration using a different random split.
  - Features were added using forward selection to maximize the accuracy of the test set.
  - The process was repeated for 10 cross-validation iterations.
  - Hyperparameters were optimized using Optuna [69], which automates the search for optimal configurations through Bayesian optimization. Each trial used a different set of hyperparameters, evaluating the R-squared score on the validation set. The best hyperparameter set for our data and model was selected after adjusting the range and re-running Optuna.

To validate the classification results, we created 100 randomized versions of the labels (permuted the labels) and ran our classification models.

### The selected features

To identify the most meaningful features, we utilized and combined several methods:

1. Frequency of features in cross-validation: we aggregated all selected features from the cross-validation runs and calculated their frequency, focusing on the top 10% most frequent features. It is important to note that we also reduced the number of features following the application of our algorithms:
  - Folding energy: we combined windows that are within 10 units of each other.
  - Sequence features: we merged features that have only a single mutation difference.
2. XGBoost feature importance: we ranked the importance of features using the XGBoost algorithm, which evaluates feature importance based on the F score [71]. The F score combines precision and recall assessing the accuracy of a binary classification model.
3. SHAP values [72]: SHAP values were used to explain the output of the machine learning model. This method employs a game-theoretic approach to quantify each feature’s contribution to the model’s predictions.

This comprehensive approach ensured a robust identification of the most significant features influencing the model’s performance.

### Transcription factors analysis

We analyzed 97 position-specific scoring matrices (PSSMs) corresponding to *E. coli* transcription factors (TFs) and calculated their scores in the original promoter sequence. To evaluate statistical significance, we generated randomized versions of the original promoter by shuffling its nucleotide sequences and computed the PSSM scores for these shuffled controls. We extracted the position with the maximum PSSM score. Using this background distribution, we calculated empirical *P*-values for the maximum PSSM score, identifying motifs that were significantly enriched. We performed this analysis for the control biosensor and our variants.

## Results

### Optimization of the *yqjF* gene promoter

An optimization algorithm was employed to enhance the DNT-sensing capabilities of the whole-cell biosensor. The procedure targeted the sensing element, the C55 version of the *yqjF E. coli* gene promoter [30, 73], located upstream to the plasmid-borne *Aliivibrio fischeri* bioluminescence *luxCDABE* gene cassette (Fig. 1A). These genes encode five proteins, two of which (*luxAB*) code for the formation of the two subunits of the luciferase enzyme, and the *luxCDE* gene products are responsible for substrate formation and recycling. The computational optimization algorithm was comprised of several sequential steps, as detailed in the “Methods” section and illustrated in Fig. 1B and C. These steps involved analyzing both endogenous and heterologous promoter data under conditions of exposure and non-exposure to DNT. Subsequently, sequence motifs that were extracted to distinguish between motifs in genes that were either overexpressed or under-expressed due to DNT exposure, utilizing a motif-finding algorithm. The algorithm then computes the similarity of motifs between the two datasets to assess the robustness of the DNT effect. It also examined these motifs’ positioning to identify the optimal insertion site within the optimized promoter based on improvements in the PSSM. Finally, the algorithm determines the final motif sequence by improving the PSSM score.

After this iterative process, a library comprising 397 variants of the *yqjF* gene promoter sequence was designed, out of which 367 plasmids were successfully synthesized and transformed into *E. coli* (Supplementary Table S1).

### Evaluating the DNT detection of the biosensor

The new biosensor strains thus generated were exposed to DNT (5 mg/l) for 10 h, and the maximum luminescence difference in the presence or absence of DNT was recorded (Fig. 2A). Notably, 197 variants (over 53%) exhibited higher maximum luminescence values compared to the unmodified parental strain (illustrated by the purple line). Luminescence development by the top five performers in response to DNT exposure is depicted in Fig. 2B; all five variants consistently outperformed the unmodified control throughout the experiment: the luminescent response started practically at time zero, compared to approximately a 2.5 h lag in the control, and maximal signal intensity was ca. fourfold higher. These findings underscore the significant enhancement in the biosensor’s ability to detect explosive materials through the utilization of computational models to optimize gene expression.

To evaluate the effect of the *yqjF* promoter sequence modifications on DNT detection sensitivity, the top five variants were exposed to a series of DNT concentrations, along with the unmodified control. As depicted in Fig. 2C, luminescence in the presence of all DNT concentrations was higher in the new variants; this was particularly apparent at the lower concentrations.

To quantify these differences in sensitivity, the DNT detection threshold was estimated by calculating the EC_200_ value, denoting the DNT concentration eliciting a bioluminescent response that is twofold higher than the uninduced control. The EC_200_ values (Fig. 2D) clearly demonstrate the enhanced detection sensitivity endowed by all five modified promoters. The improvements in both detection threshold and signal intensity are summarized in Fig. 2E, in which luminescence intensity at the EC_200_ DNT concentration is plotted as a function of the EC_200_ value. To further assess the statistical significance of expression changes, we performed a two-sample *t*-test comparing the maximal luminescence difference values of each variant to the control variant. Each comparison was based on two experimental replicates. This analysis yielded 144 variants with statistically significant differences (*P* < .05), indicating that a substantial subset of the designed sequences led to meaningful alterations in expression levels relative to the control (when we randomized the data, the maximum significant variants are 49, and on average, only seven variants are significant), suggesting an FDR of 4.9%. We performed the same analysis for all variants with each other (significant results can be seen in Supplementary Fig. S7). On average, each variant is significantly different from 25 other variants.

### Evaluating the top variants’ responses to DNT in realistic conditions

**Figure 3.**
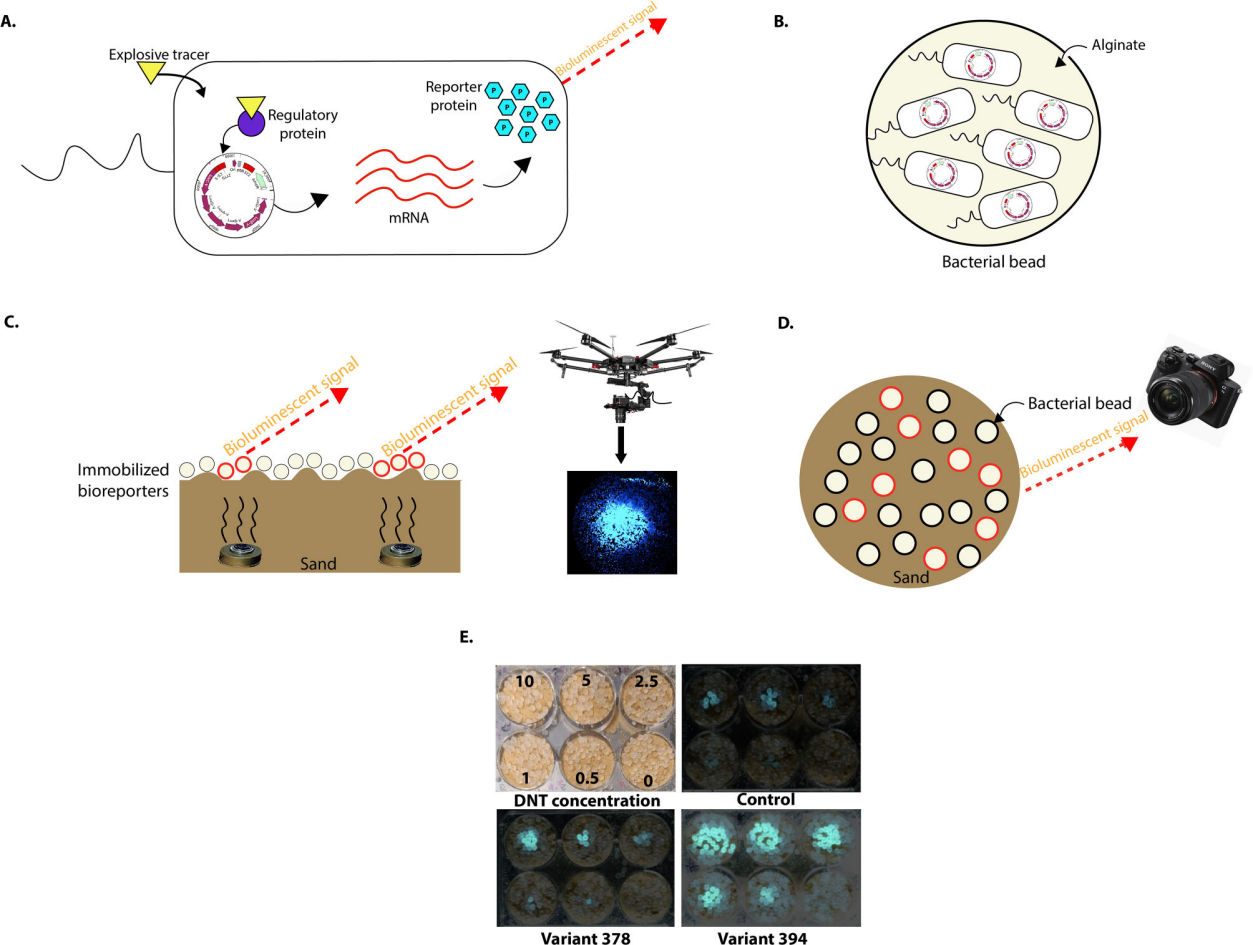
(**A**) Biosensor reaction to DNT illustration. (**B**) Bacterial beads illustration. (**C**) Real-life experiment illustration. (**D**) Experiment illustration. (**E**) Bioluminescence of alginate-encapsulated strains 394 (D), 378 (C), and the control (C55) (B) following a 3-h exposure to various DNT amounts on sand. Each well of a six-well microtiter plate harbored monolayer beads (A). The plates were incubated at room temperature and imaged in a dark chamber at 20-min intervals.

To further assess the performance of our variants, we identified two top-performing candidates based on their high luminescence levels and sensitivity, as illustrated in Fig. 2E (the biosensor response to DNT is schematically shown in Fig. 3A). These variants were subsequently evaluated under conditions mimicking real-world scenarios. The biosensors were encapsulated in alginate beads (Fig. 3B) and placed on sand at room temperature in complete darkness (Fig. 3C and D schematically illustrate the experimental setup and a comparative laboratory experiment). As shown in Fig. 3E, both variants outperformed the control biosensor, demonstrating superior luminescence and enhanced sensitivity across all tested DNT concentrations. While the images in Fig. 3E were recorded 3 h after exposure of the bacterial beads to the DNT-containing sand.

### Computational analysis of the variants

**Figure 4.**
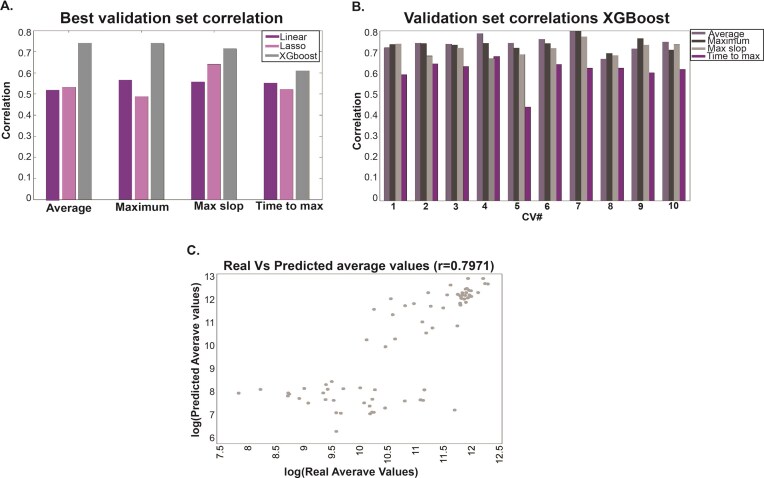
Prediction algorithm results. (**A**) Best cross-validation, validation set correlation for all predicted variables. (**B**) XGBoost validation set correlation for all cross-validation and predicted variables. (**C**) Dot plot of the real and predicted variable “Average value.”

Three models were employed to analyze the predictive variables, as described in the “Methods” section: Linear regression, Lasso (Least Absolute Shrinkage and Selection Operator), and XGBoost (eXtreme Gradient Boosting). Figure 4A presents the cross-validation results, highlighting XGBoost as the model with the highest correlation in the validation set. Subsequent analyses were therefore based exclusively on this model.

Figure 4B illustrates the correlation results for all cross-validation iterations across the different predictive variables, and Fig. 4C displays the scatter plot for the variable “Average value.” Additional data are provided in the Supplementary Materials.

The results indicate that our algorithm can predict the main variables with high precision. Even when correlating the two repeats of the experiment, a correlation of 0.83 was achieved, demonstrating the robustness of our predictive model.

**Figure 5.**
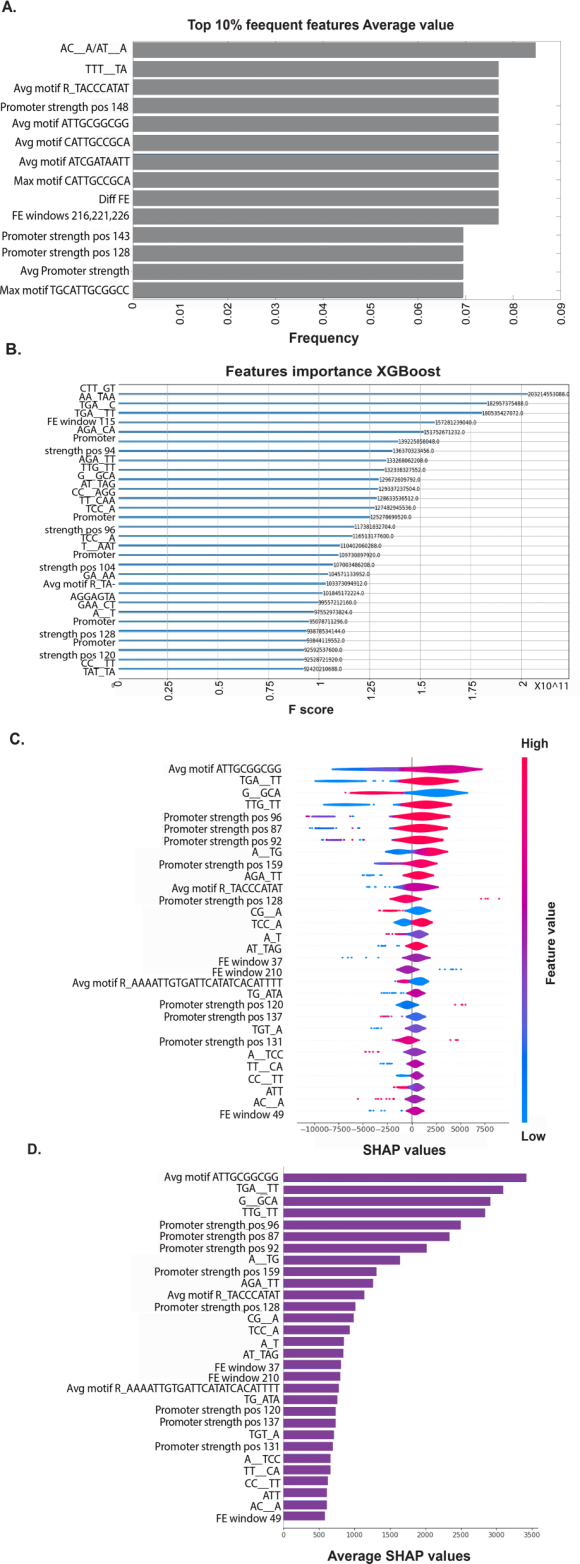
Most influential features predictor analysis. (**A**) Top 10% of frequent features from all cross-validation sets. (**B**) Top 30 features F score. (**C**) Top 30 features SHAP values and direction. (**D**) Top 30 features averaged SHAP values.

Given the strong correlation obtained, the next step was to analyze the features contributing to these results for each of the predictive variables. Figure 5 represents the “Average value” variable, and all other variable analyses can be seen in Supplementary Figs S8, S9, and S10. First, we calculated the frequency of appearance of all features across all cross-validation iterations (Fig. 5A, highlighting the top 10% of frequent features). Second, the XGBoost algorithm was utilized to rank the importance of features based on their F scores [71] (Fig. 5B, presenting the top 30 features). Third, we employed SHAP (SHapley Additive exPlanations) [72] values to identify the most influential features and their direction of influence (Fig. 5C and D, showcasing the top 30 features). For more detailed information, see the “Methods” section.

From this analysis, we observed that the most influential features were DNA folding in different windows, sequence motifs, and promoter strength. These findings are consistent with biological expectations: folding affects the ability to transcribe the coding sequence [74–76], while sequence motifs and promoter strength are crucial for the control and recruitment of TFs [77]. Notably, different techniques highlighted different important features, which is expected, as each method considers different aspects of feature importance [78, 79]. For example, when looking at our analysis of the frequency of features, it is affected by the cross-validation data division, while the SHAP values analysis focuses on the local and spatial explanations of the features; therefore, different results can be obtained.

We have also correlated all important features identified by the various techniques to determine their consistency (Supplementary Fig. S11). We found that 50% of these features exhibited a high degree of correlation, indicating that they were robustly selected across different methods.

**Figure 6.**
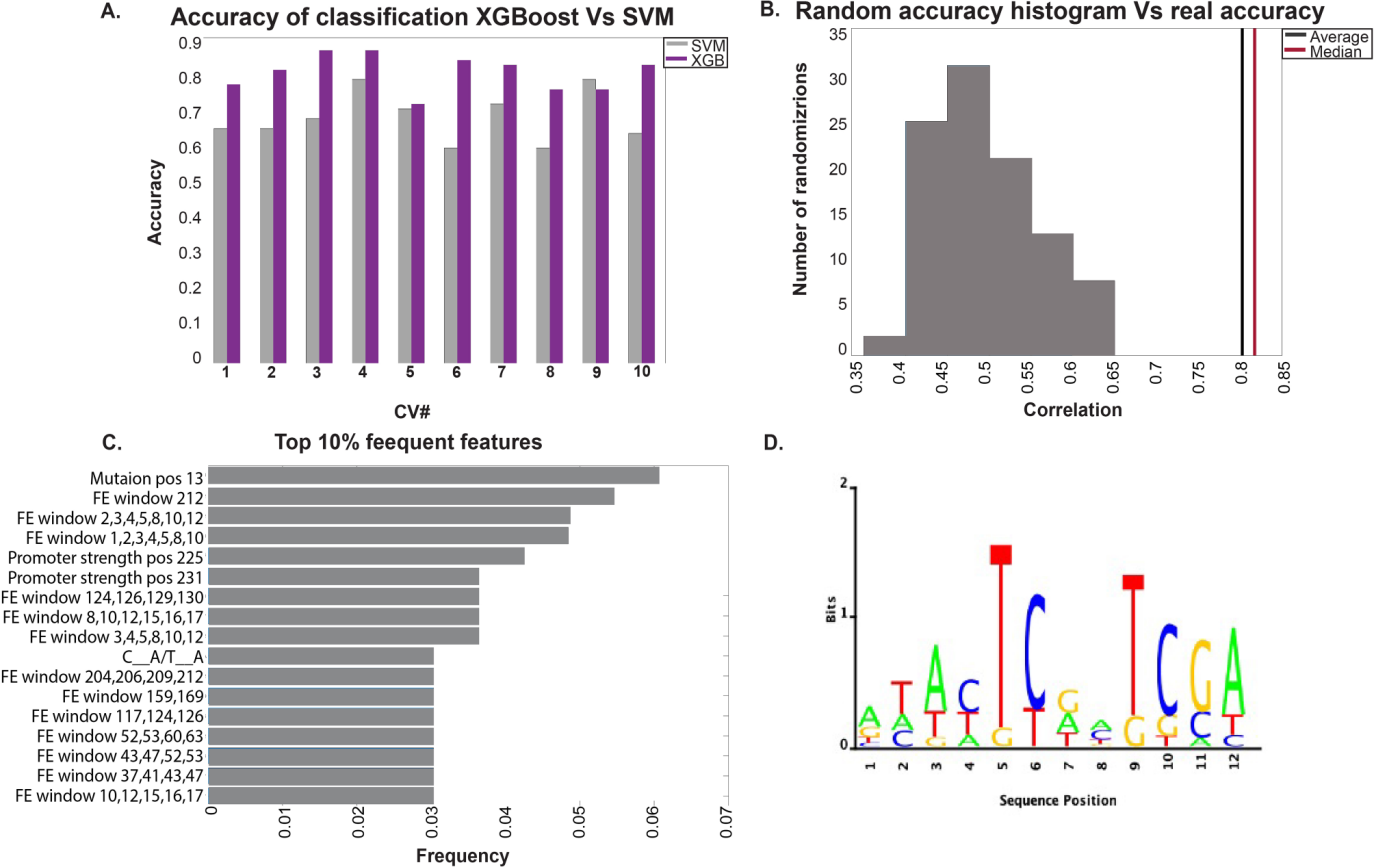
Classification algorithm and TFs analysis results. (**A**) XGBoost and SVM accuracy in all cross-validation sets. (**B**) Random accuracy distribution and real accuracy average and median of all cross-validation sets. (**C**) The top 10% frequent features of the classification; feature labels are detailed in the “Methods” and “Supplementary” sections. (**D**) Sequence logo of the significant motif.

As shown in Fig. 2A, the maximum luminescence difference differed significantly between the clones. Specifically, 53% of the variants exhibited higher values than the unmodified variant, while a substantial number of clones showed differences close to zero (indicating no observable change with and without DNT). This observation led us to create a classification algorithm to understand the reasons for this meaningful observation. The accuracy values obtained by employing the XGBoost and SVM (support vector machine) classifiers from all cross-validation iterations are shown in Fig. 6A. The results were robust, with XGBoost achieving an average accuracy of 0.8027, outperforming SVM, which had an average accuracy of 0.6784. Consequently, subsequent analyses were focused on XGBoost.

To validate the accuracy, we permuted the labels 100 times and reran the algorithm, calculating the accuracy for each randomization run. Figure 6B demonstrates that the classification accuracy with real labels was higher than all random accuracies.

We then examined the selected features by identifying the most frequent features across all cross-validation sets (Fig. 6C, top 10% of frequent features). Notably, folding features had the most significant influence on the differences in maximum luminescence between variants.

Recent strategies in optimizing the DNT-sensing biosensor have moved beyond molecular modifications of the yqjF promoter and its regulator YhaJ, instead leveraging computational approaches to systematically enhance sensor performance. YhaJ is a central TF in *E. coli* responsible for modulating the yqjF promoter in response to environmental signals, particularly metabolites of explosives like DNT. Historically, molecular modifications, including directed evolution or mutagenesis of the yqjF promoter and YhaJ, were used to incrementally boost sensor sensitivity. However, these approaches often did not address whether sequence-level features specific to YhaJ binding motifs, such as the presence or absence of PSSM motifs, differences between variant and control strains [80–82].

Therefore, we specifically aimed to assess whether the control strain and our engineered promoter variants contained the PSSM motif recognized by YhaJ. We examined the score of the PSSM matrix of the TF YhaJ across our variants. Comparing the top and bottom 20% based on luminescence difference, the Wilcoxon test yielded a non-significant result (*P* = .4348), suggesting that, in our experiment, YhaJ does not directly affect performance.

In addition to evaluating the presence of the YhaJ motif, we also systematically screened for other TFs [61] that could significantly influence biosensor performance in both control and variant promoter sequences (“Methods” section). This broader search enabled the identification of additional regulatory sequences and potential TF-binding sites enriched in the engineered promoter variants compared to the control, such as FhIA, GadW, GlpR, NarL, and ArgR (Supplementary Tables S4 and  S5; Supplementary Fig. S12).

To try to identify new TF motifs, we analyzed the 12 new motifs that were identified (“Methods” section), focusing on the top and bottom 20% of variants ranked by their maximum luminescence difference. For each variant, we calculated its maximum PSSM score and compared the two groups using the Wilcoxon test. This analysis revealed that one motif showed a statistically significant correlation, and its sequence logo is presented in Fig. 6D.

## Discussion

In this study, we applied a computational-synthetic biology approach to improve the performance of a DNT biosensor, creating 367 variants that were evaluated against an existing strain [52]. Prior strategies largely focused on molecular modifications, such as the directed evolution of the yqjF promoter [8] and its regulator YhaJ [80], as well as beneficial mutations introduced into the host bacterium [23]. Recently, additional manipulations of the YhaJ regulator [81] have further improved DNT detection capabilities. In the present communication, we highlight a different approach for sensor strain improvement, based on a computational approach that aims to identify motifs and features from sequences of variants or endogenous genes both exposed and unexposed to an explosive and to infer which specific subsequences are affected and the reasons behind their influence. The result was significant, with 53% of the new variants exceeding the original strain’s performance, demonstrating this approach’s value.

The computational tools enabled the identification of crucial features affecting biosensor performance. DNA folding patterns emerged as critical to regulatory element accessibility, potentially allowing TFs to bind more efficiently. Additionally, specific nucleotide motifs influenced biosensor sensitivity by functioning as key recognition sites for TFs. These findings point to the broader significance of integrating computational techniques with synthetic biology to improve biosensor design. Beyond the specific application of explosive detection, the design principles and computational framework presented in this study are broadly applicable to other biosensing challenges. The modularity of promoter-reporter systems, combined with machine learning-based motif optimization, enables the tailoring of biosensors for diverse environmental, industrial, or clinical targets. With access to relevant expression datasets, such as transcriptomic responses to a specific analyte, it becomes not only feasible but relatively straightforward to adapt the system to detect alternative compounds. This process involves identifying enriched sequence motifs and regulatory elements responsive to the new target, which can then be computationally optimized and inserted into the biosensor chassis. Similar strategies have already been employed to engineer microbial sensors for detecting heavy metals, endocrine disruptors, and disease-related biomarkers [14, 17, 18, 83]. The ability to fine-tune sensitivity, response time, and signal strength through predictive modeling makes this approach particularly valuable for developing multiplexed biosensing platforms capable of detecting multiple analytes simultaneously in complex environments [15, 20]. Moreover, as synthetic biology expands into diagnostic and therapeutic domains, whole-cell biosensors optimized through computational methods may serve as a foundation for intelligent living diagnostics [43, 51], or as embedded quality control tools within biomanufacturing systems [38]. These findings highlight not only the versatility of our approach but also its potential to accelerate innovation across biosensor applications.

Endogenous transcripts are shaped by complex evolutionary constraints, making it difficult to isolate the effect of specific sequence features on gene expression [44, 46, 47]. These factors, such as overlapping regulatory signals, codon bias, and native chromosomal context, limit the ability to draw clear causal conclusions. In contrast, our synthetic biology approach, based on large-scale libraries of systematically mutated promoter variants, offers a controlled experimental framework. By varying one or a few features at a time in an otherwise constant background, this method enables precise dissection of how elements like DNA folding patterns, motif position, and promoter strength influence biosensor output. As demonstrated here and in other studies [48–50, 80], synthetic constructs allow clearer causal inference and robust identification of functionally significant design principles.

Our experiment and analysis suggest that DNA folding features play a major role in determining biosensor performance, likely by affecting TF access or polymerase binding. This hypothesis can be further experimentally validated by introducing targeted mutations in promoter regions that alter predicted folding structures while keeping key binding motifs intact. By designing variants that specifically adjust folding without disrupting regulatory sequences, we can compare biosensor outputs and directly assess the impact of folding. Constructing folding-energy gradient libraries and measuring reporter gene activity in high-throughput screens would further clarify how folding influences sensitivity and output. Future studies using these approaches could confirm DNA folding as a critical and tunable parameter in synthetic promoter engineering.

The performance of our engineered DNT biosensor variants was assessed using a high-throughput luminescence reporter assay, enabling direct measurement of transcriptional output from synthetic promoters, with all coding regions held constant across variants. This approach allowed for systematic evaluation of hundreds of variants, a scale not feasible with qPCR, and aligns with widely accepted methods for assessing promoter strength and biosensor activity in the literature. Our results showed that top DNT-responsive promoter variants produced significantly higher luminescence than parental and control strains, in line with established benchmarks for whole-cell biosensor performance.

Furthermore, this study opens the door to a deeper exploration of the metabolic pathway involved in DNT sensing. While both DNT and TNT activate the yqjF promoter, the data suggest that their degradation product, trihydroxytoluene [84], is the more likely inducer. This metabolic interaction, combined with the role of YhaJ in regulating yqjF activation [82], offers a compelling opportunity for further optimization of the biosensor [81], focusing on enhancing the biosensor’s sensitivity and performance by targeting these regulatory mechanisms. The significant performance improvements of the computationally optimized biosensor variants under realistic conditions, such as testing on sand (as in real life) at room temperature in the dark - further highlight the practical relevance of these findings. The variants demonstrated not only superior luminescence but also enhanced sensitivity across all tested DNT concentrations, reinforcing the potential of this approach to advance biosensor technology.

While previous studies have successfully improved whole-cell biosensors through random mutagenesis or library-based screening [85, 86], these approaches typically rely on extensive experimental screening and lack mechanistic insight into the contribution of specific sequence features. In contrast, our study employs a rational, computationally guided strategy that models gene expression outputs based on promoter sequence and structural features. By incorporating machine learning to predict luminescence and integrating DNA folding and motif analyses, we were able to directly link promoter design to biosensor performance. This allowed us to rapidly identify high-performing variants without exhaustive library screening, thus streamlining development and enabling a deeper understanding of functional determinants, particularly folding features and motif arrangements. To our knowledge, this is the first application of such modeling-driven promoter design in the context of yqjF-based explosive biosensors, resulting in improved dynamic range and sensitivity compared to prior designs [8] (Supplementary Table S6).

By integrating metabolic insights with the optimization of regulatory interactions using computational analysis, future developments can create even more robust and versatile biosensors suitable for diverse real-world applications.

## Conclusions

In conclusion, this study demonstrates the substantial potential of computational approaches for optimizing biosensors. By analyzing structural and sequence motifs in newly engineered variants, we identified critical features such as DNA folding patterns and specific nucleotide motifs that significantly enhanced biosensor performance. Importantly, our computationally designed biosensor variants outperformed those produced using traditional molecular modification strategies, with 53% exceeding the original strain’s performance. This highlights the value of integrating computational models to predict and fine-tune regulatory elements underlying biosensor function.

Our findings further show that these optimized variants maintain superior sensitivity and luminescence under realistic testing conditions, including solid matrix assays at room temperature and in darkness, consistently surpassing control strains across all tested DNT concentrations. These results emphasize the reliability and sensitivity of these biosensors for practical environmental and security applications.

The broader applicability of this strategy is clear, as the underlying computational and synthetic biology framework can be adapted to diverse biosensing challenges, from environmental monitoring to industrial and medical diagnostics. The modular and scalable nature of the approach, paired with machine learning-based motif optimization, allows precise tuning of sensor properties for different targets.

Additionally, insights into the metabolic pathway of DNT sensing, especially the potential role of trihydroxytoluene as an inducer, underscore the need for further research into optimizing these metabolic interactions. Future directions should focus on enhancing sensor performance by refining the regulatory interplay between engineered promoters and the YhaJ TF, as well as leveraging metabolic pathway understanding for improved sensitivity. Overall, this work establishes a robust foundation for future innovations in biosensor technology, reaffirming the powerful synergy between synthetic biology and computational modeling for solving complex biological sensing problems.

## Supplementary Material

gkaf1482_Supplemental_Files

## Data Availability

All data generated or analyzed during this study are included in this published article [and its supplementary information files].
