# Supplementary material for: Design of bacterial DNT sensors based on computational models
Source: Nucleic Acids Res. 2026 Jan 8;54(1):gkaf1482. doi: 10.1093/nar/gkaf1482 (PMC12781885; doi:10.1093/nar/gkaf1482)
Supplement: gkaf1482_Supplemental_Files [file gkaf1482_supplemental_files.zip › Supplementary_DNT_new.pdf]

Supplementary:

Supplementary table S1 – Table of all generated variants. Sperate file.

Supplementary table S2 – Table of the synthetic library data. Sperate file.

Supplementary table S3 – Table of all features names and description

Feature Name	Feature Description
FE window i	Folding energy in a window of 40 nt starts with nt i
Diff FE	Average folding of all windows
Total FE	Folding energy of the entire variant
Mut i	Binary feature 1/0 if a mutation axist or not in position i
Mutation amount	The amount of mutation in the variant
cARS	Chimera ARS score
Max_motif_X	Maximum PSSM score of motif X from the extraction of new motifs as described in the Sequence Motifs Extraction section.
Avg_motif_X	Average PSSM score of motif X from the extraction of new motifs as described in the Sequence Motifs Extraction section.
W motif	Maximum PSSM Scores inserted motifs from data set B.
E motif	Maximum PSSM Scores inserted motifs from data set A.
Max motif X Regulon	Maximum PSSM score of motif X from SwissRegulon
Avg motif X Regulon	Avergae PSSM score of motif X from SwissRegulon
Avg promoter score	
	Average Promoter Strength: Overall promoter strength across the entire sequence

Promoter strength pos i	Strength by Position: Promoter strength calculated for specific positions i	
zCurve	x_axis =$(\Sigma A+\Sigma G)-(\Sigma C+\Sigma T)$	
	y_axis =$(\Sigma A+\Sigma C)-(\Sigma G+\Sigma T)$	
	z_axis =$(\Sigma A+\Sigma T)-(\Sigma G+\Sigma C)$	
gcContent	$(\Sigma G+\Sigma C)/(\Sigma A+\Sigma C+\Sigma G+\Sigma T) * 100\%$	
ATGC ratio	$(\Sigma A+\Sigma T)/(\Sigma G+\Sigma C)$	
Cumulative Skew	GC Skew=$(\Sigma G-\Sigma C)/(\Sigma G+\Sigma C)$	
	AT Skew=$(\Sigma A-\Sigma T)/(\Sigma A+\Sigma T)$	
Pseudo KNC	Features will be numbers of A, C, G, T, AA, AC, AG, AT, CA, CC, CG, CT, GA, GC, GG, GT, TA, TC, TG, and TT of the whole sequence of DNA respectively.	
monoMonoKGap	Features will be numbers of A_A, A_C, A_G, A_T, C_A, C_C, C_G, C_T, G_A, G_C, G_G, G_T, T_A, T_C, T_G, T_T, A__A, A__C, A__G, A__T, C__A, C__C, C__G, C__T, G__A, G__C, G__G, G__T, T__A, T__C, T__G, T__T of the whole sequence of DNA respectively.	
monoDiKGap	Feature structure will be X_XX, and X__XX of the whole sequence of DNA respectively.	
monoTriKGap	Feature structure will be X_XXX, and X__XXX of the whole sequence of DNA respectively.	
diMonoKGap	Feature structure will be XX_X, and XX__X of the whole sequence of DNA respectively.	
diDiKGap	Feature structure will be XX_XX, and XX__XX of the whole sequence of DNA respectively.	
diTriKGap	Feature structure will be XX_XXX, and XX__XXX of the whole sequence of DNA respectively.	
triMonoKGap	Feature structure will be XXX_X, and XXX__X of the whole sequence of DNA respectively.	
triDiKGap	Feature structure will be XXX_XX, and XXX__XX of the whole sequence of DNA respectively.	
SD i	Ribosome binding site strength position i	

Supplementary table S4 – Significant transcription factors and their binding motifs were identified in the control strain promoter, highlighting potential regulatory sequences relevant for DNT sensing performance.

TF motif name	Seqlogo	Position (nt) from the 5' end
**FhlA**	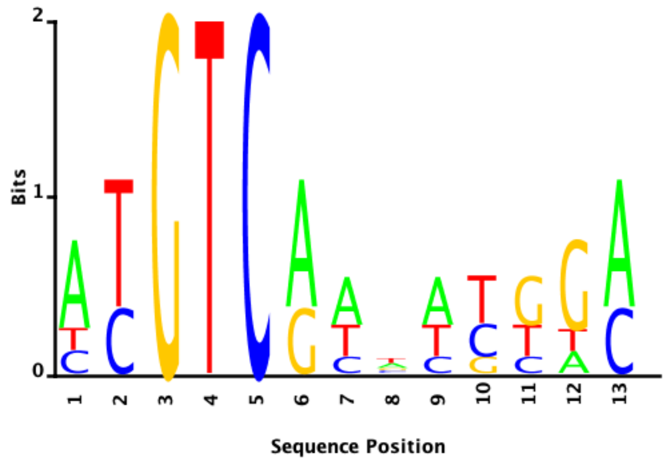	**369**
**GadW**	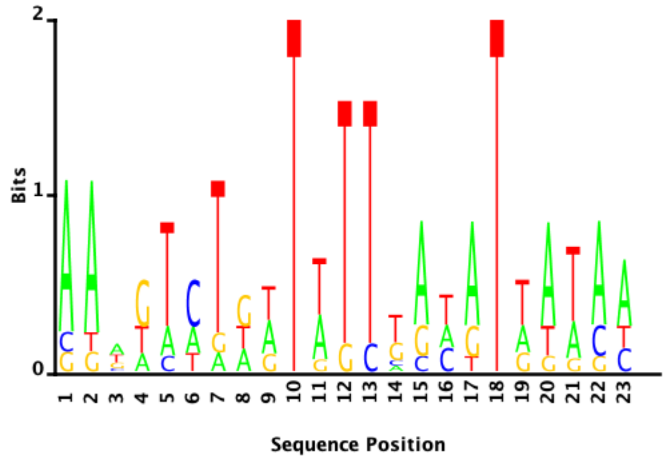	**268**

**GlpR**	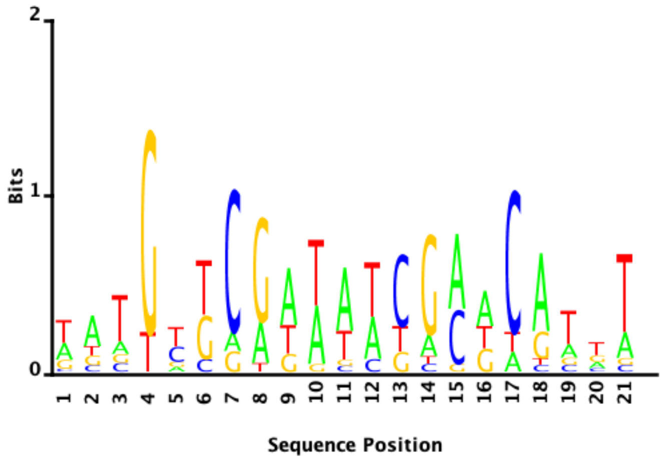	**381**
**NarL**	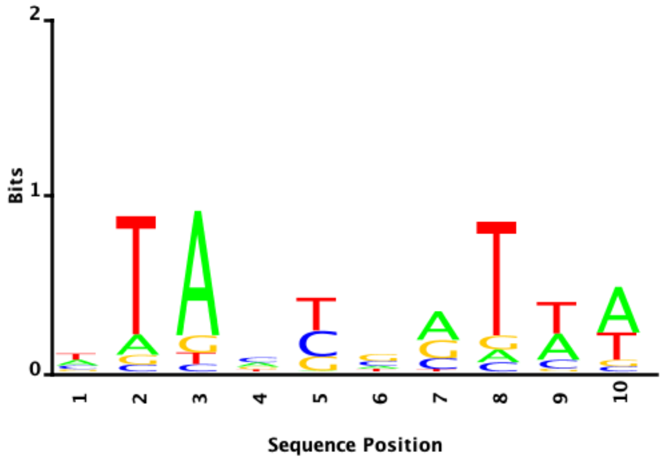	**345**
**ArgR**	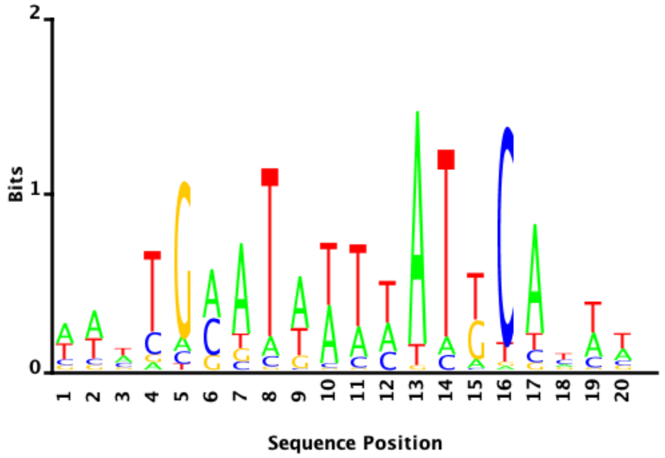	**309**

Supplementary table S5 – Table of significant TF motifs in all the variants

TF motif ID	TF motif name	Number of significant variants
1	FhIA	8
6	GadW	8
9	GalS	18
13	GlrR	156
14	GntR	367
16	HipAB	367
17	HipB	367
26	MelR	366
28	MetR	367
29	MiC	367
31	ModE	367
32	MqsA	24
35	NagC	291
36	NanR	79
39	NarP	91
40	NhaR	367
41	NrdR	360
45	PdhR	190
48	PlaR	367

49	PurR	331
50	PutA	132
51	PuuR	367
53	RacR	366
54	RbsR	367
57	RelBE	31
59	Rob	314
60	RutR	367
61	SdiA	367
67	TrpR	367
69	UiaR	367
71	YdeO	264
73	Ada	367
80	BaeR	367
81	BasR	27
83	CaiF	367
88	CysB	367
91	DcuR	294
94	EvgA	367
96	FeaR	367

Supplementary table S6 – Table of comparison with previous studies

Study	Organism	Target Compound	Promoter Design Strategy	Further approach explanation	Reporter Gene	Signal Type	Key Advantage	Performance Results
This Study	E. coli	DNT/TNT	Computational modeling of gene expression, motif analysis, DNA folding	analyzed motifs across the E. coli UTR's and synthetic promoters library	luxCDABE	Bioluminescence	High performance with minimal screening; design and study of features that haven't been studied before; mechanistic insight via comprehensive modeling	2–4× luminescence increase vs. C55; targeted design, low screening
Chen et al. 2022	E. coli	As(III)	De novo synthetic promoter library informed by ArsR binding sites	The study used a de novo synthetic promoter library informed by the known ArsR transcription factor binding site. The library included thousands of randomized sequences flanking the binding motif to capture diverse expression strengths.	GFP	Fluorescence	High sensitivity via large-scale experimental screening	Detection limit: 0.02 µg/L As(III); >1000 variants screened
Valenzuela-García et al. 2023	B. subtilis (Spores)	As(III), As(V)	Native Pars promoter with spore-based platform	This group used a native promoter, pars, from B. subtilis that is naturally regulated by arsenic species. Instead of engineering the promoter, they embedded it in a spore-based biosensor system. The key innovation was in packaging and deployment, rather than sequence-level design.	GFP	Fluorescence	Long shelf-life, stable biosensor via spores	Moderate sensitivity; robust spore-based delivery
Hernández-Sancho et al. 2024	P. putida	Various chemicals	Synthetic auxotrophy (growth-coupled system)	Developed a growth-coupled biosensing system using P. putida auxotrohy. Instead of linking signal to a transcription factor or promoter activity, biosensor function was tied to the survival or growth of the bacterium	msfGFP	Fluorescence	Flexible platform independent of TF-specific activation	Growth-based output; adaptable to various targets
Yagur-Kroll et al. 2015	E. coli	DNT/TNT	Mutated yqjF promoter (C55 variant)	This work introduced point mutations into the native yqjF	luxCDABE	Bioluminescence	Benchmark design;	Baseline bioluminescence reference (C55)

				promoter and screened for improved response to DNT/TNT using bioluminescence.			moderate sensitivity	
Henshke et al. 2021	E. coli	Explosives	Random mutagenesis + high-throughput screening	Applied random mutagenesis across the promoter region of an explosive-sensing system in E. coli.	luxCD ABE	Biolu minesc ence	Diverse outputs; limited mechanistic guidance	Improved clones post-screening; limited mechanistic interpretation

A.

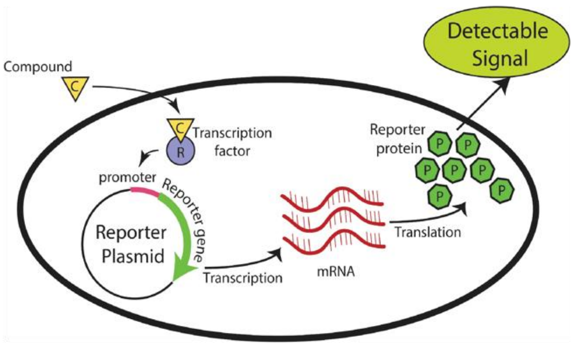

B.

# Mutations	# Sequences
1	441
2	32221
3	4901
4	4931
5	2955
6	1001
7	201

C.

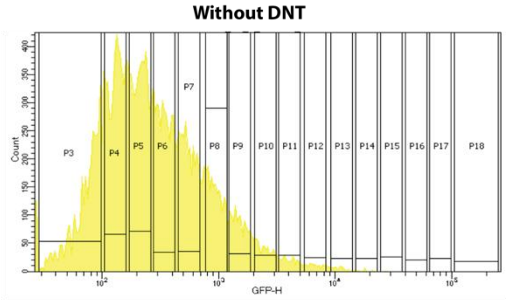

D.

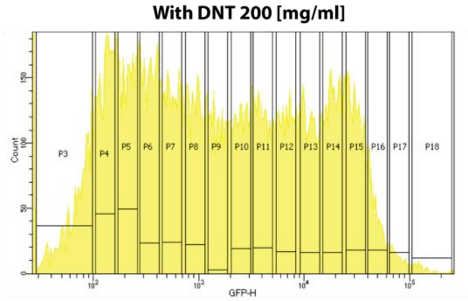

Figure S1. Synthetic library design. A. *A promoter-based biological sensor was the model system. The promoter was connected to a GFP (reporter gene) and the fluorescence levels were measured with and without exposure to DNT.* ***B.*** *The library was designed to contain all single mutations of, and a large sample of mutation pairs, triplets, and higher order mutations as can be seen in the table.* ***C.*** *The library was sorted using FACS into 16 bins (in log scale) according to GFP fluorescence. The measured GFP fluoresces without DNT.* ***D.*** *The library was sorted using FACS to 16 bins (in log scale) according to GFP fluorescence. The measured GFP fluoresces in the presence of 200* μ*g/ml of DNT.*

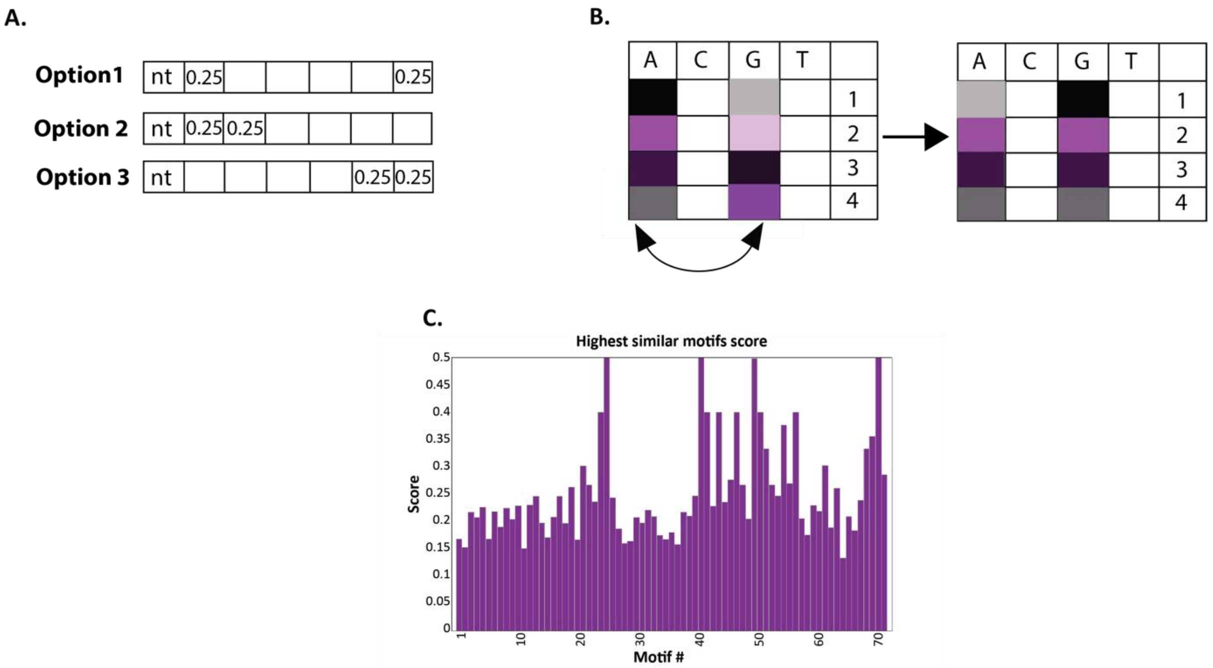

Figure S2. ***A***. *Motifs padding options. To equal the motif's dimension, we padded the edges of the relevant motifs with equal probabilities for each nt. An example in the illustration is padding a motif with four letters to a motif with six.* ***B.*** *Permutation illustration. We permuted the nt distribution of the motifs PSSM to create a null model to find significant similarity scores (Method section).* ***C.*** *We calculated a similarity score between each pair of motifs (data set A and data set B). Only significant motifs with the highest similarity score were chosen. The bar graph shows the highest similarity score of the significant motifs.*

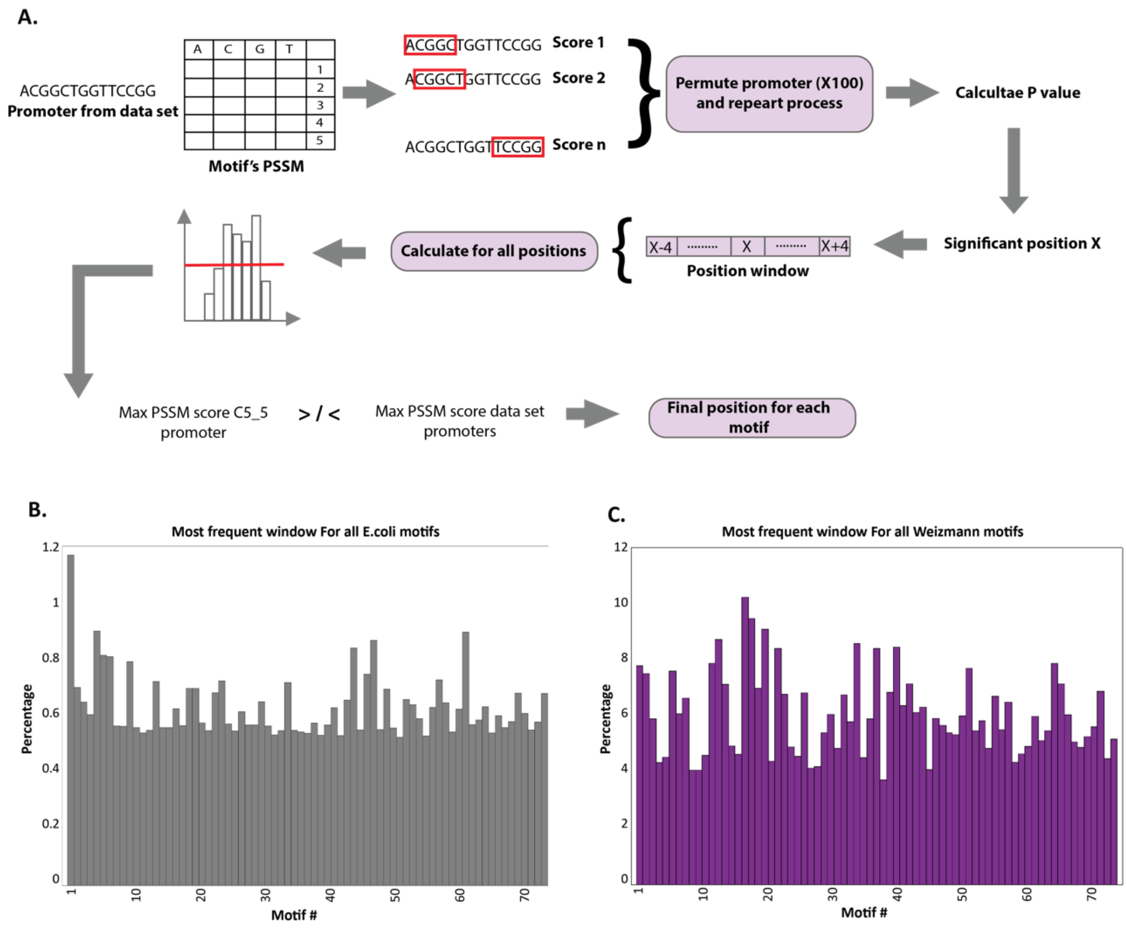

Figure S3. ***A.*** *Illustration of the algorithm that finds significant positions of the motifs.* ***B.*** *Most frequent positions window for all motifs (Methods section) data set A.* ***C.*** *Most frequent positions window for all motifs (Methods section) data set B.*

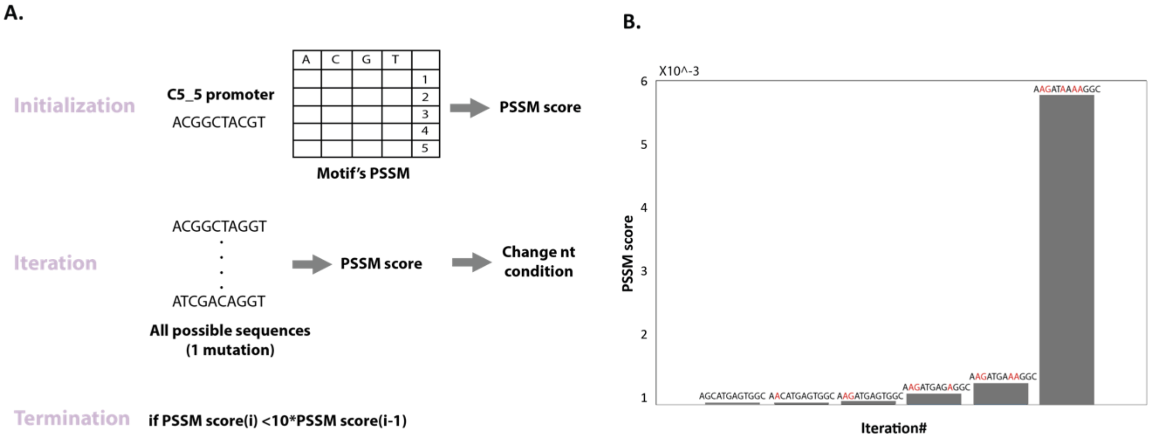

Figure S4. A. *Illustration of the algorithm that finds motif sequences (Method section).* ***B.*** *An example run of the algorithm. The graph shows the improvement of the PSSM score and the change in the nt sequence on each iteration.*

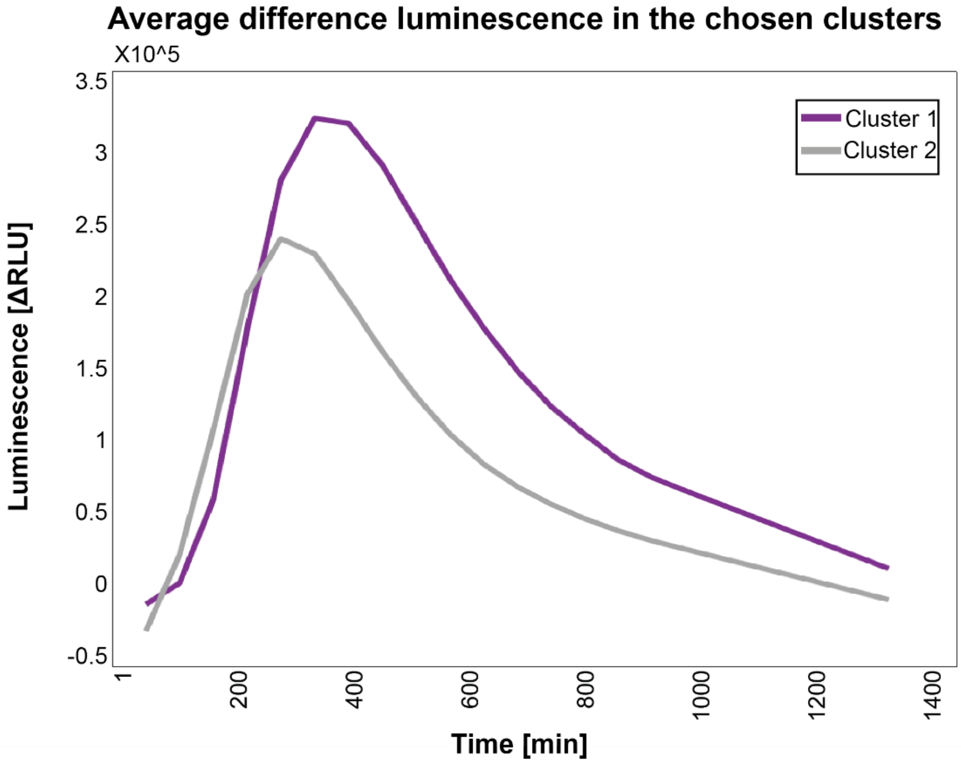

Figure S5. *All luminescence difference values of the variants in each cluster were averaged to examine different trends in the groups.*

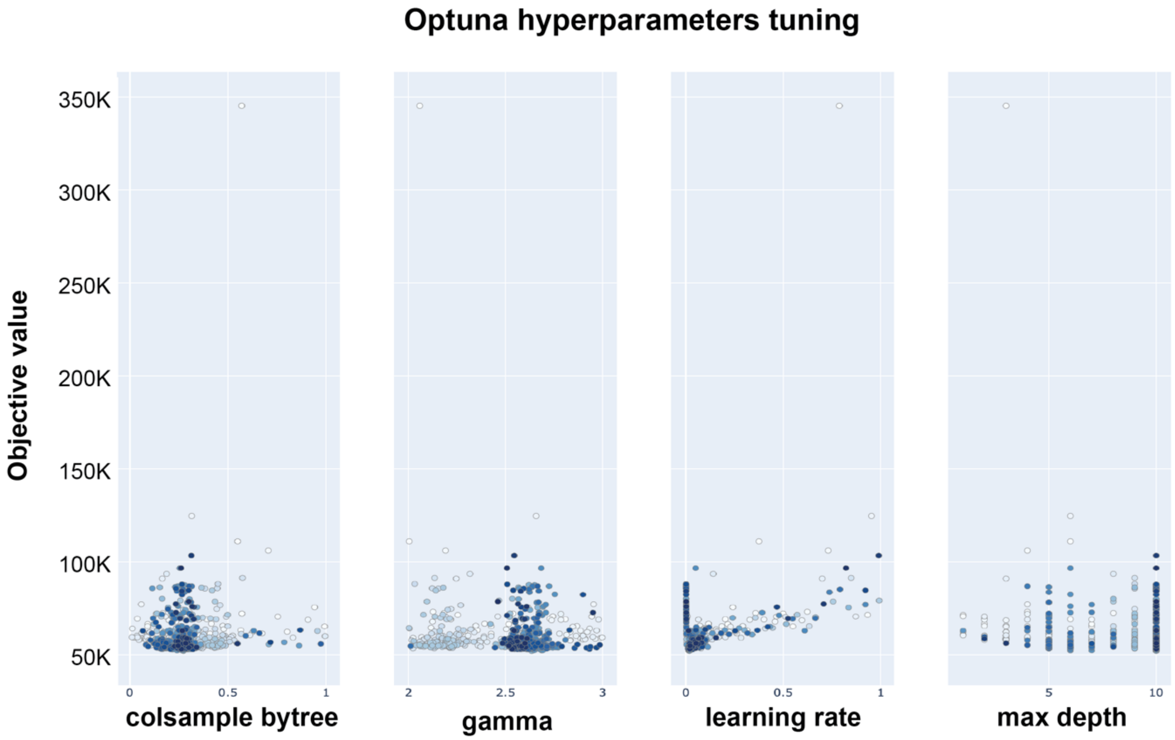

Figure S6. Hyperparameter optimization by Optuna. *The optimization of hyperparameters using Optona involved systematically testing a range of values to identify the optimal settings for each parameter. As illustrated in the graph for a few parameters, Optona evaluated various hyperparameters, demonstrating the differences in performance across the tested ranges. This process revealed that some hyperparameters had a broader range of values tested, which allowed us to assess their impact comprehensively. Based on these evaluations, it became evident that certain ranges could be narrowed down iteratively, focusing on the most promising intervals. By reducing the range and concentrating on these optimal intervals, we could more effectively pinpoint the best values for each hyperparameter, enhancing the overall model performance and efficiency.*

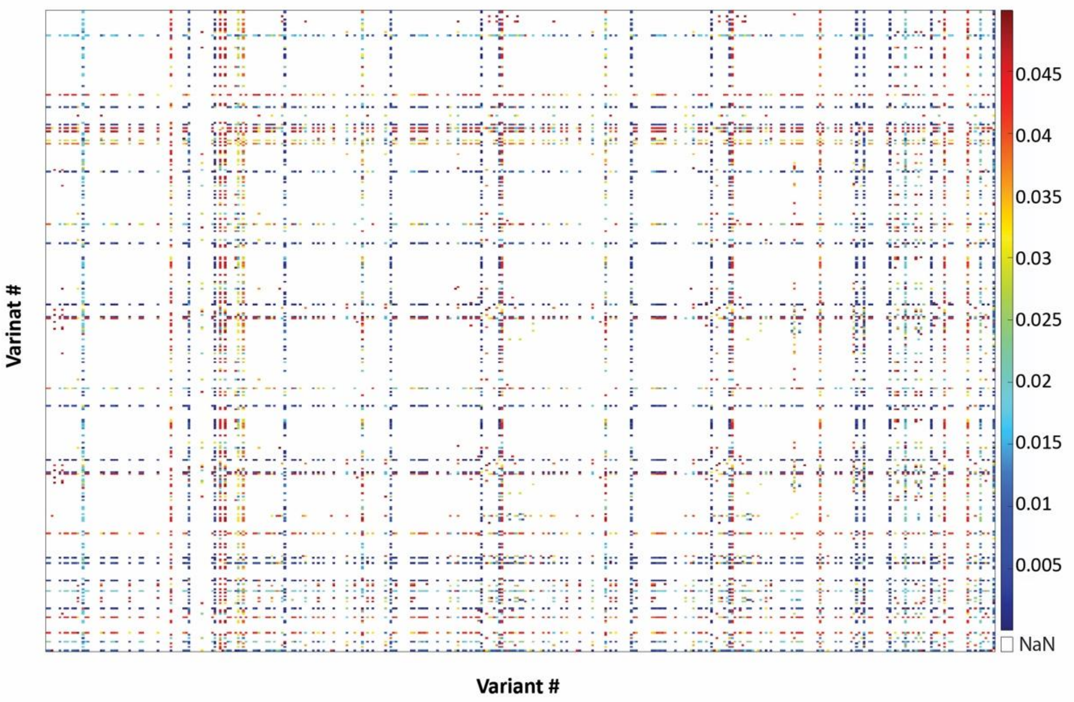

Figure S7. *Statistical analysis of maximal luminescence differences between biosensor variants. A two-sample t-test (based on two replicates per comparison) identified 144 variants showing significant expression changes relative to the control ($p < 0.05$). Randomized data resulted in an average of only 7 significant variants, indicating a low false discovery rate (4.9%). When all variants were compared to each other, each was significantly different from an average of 25 other variants. Significant pairwise differences are visualized in the accompanying heatmap.*

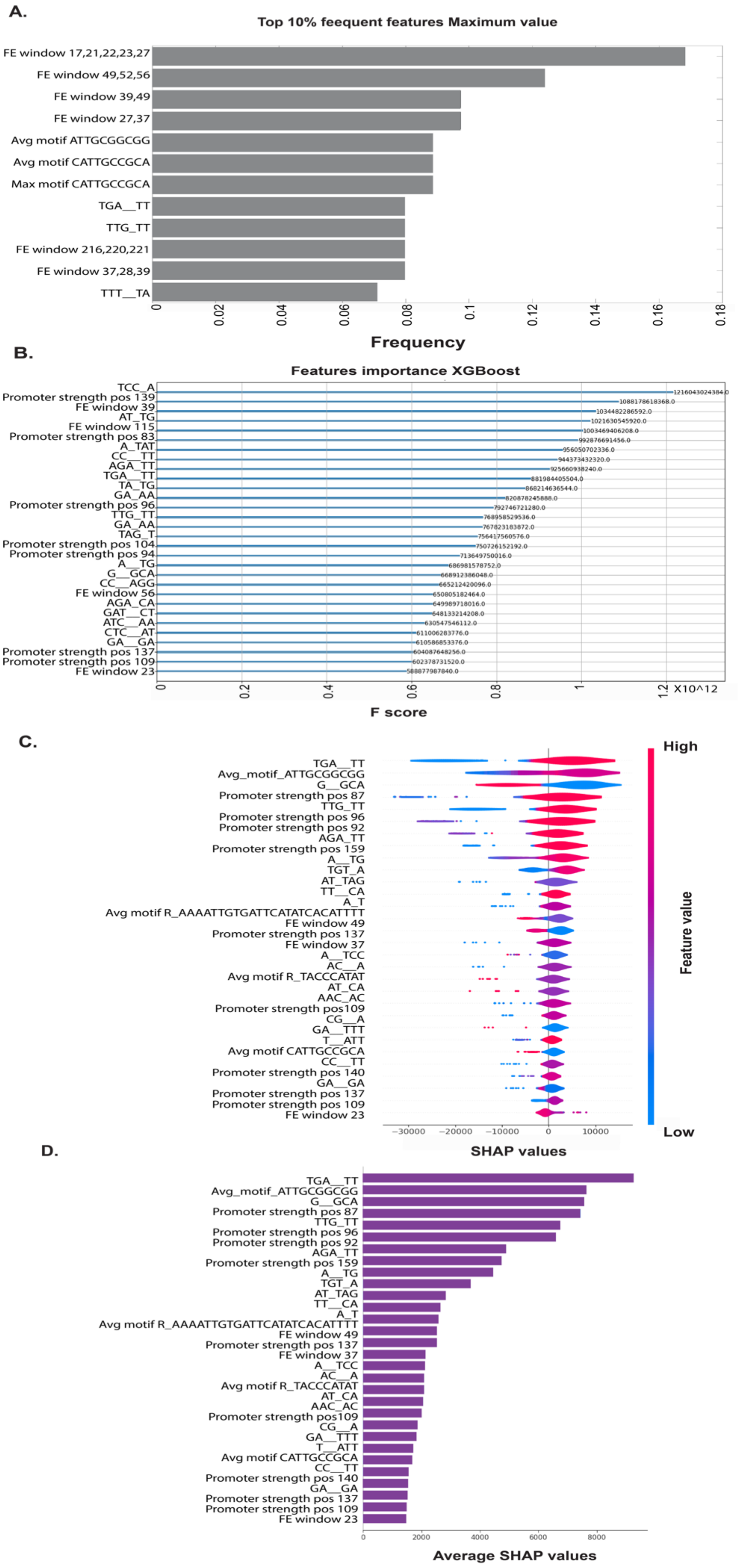
A.
Top 10% feequent features Maximum value
FE window 17,21,22,23,27
FE window 49,52,56
FE window 39,49
FE window 27,37
Avg motif ATTGCGGCGG
Avg motif CATTGCCGCA
Max motif CATTGCCGCA
TGA__TT
TTG_TT
FE window 216,220,221
FE window 37,28,39
TTT__TA
0
0.02
0.04
0.06
0.08
0.1
0.12
0.14
0.16
0.18
Frequency
B.
Features importance XGBoost
TCC_A
Promoter strength pos 139
FE window 39
AT_TG
FE window 115
Promoter strength pos 83
A_TAT
CC__TT
AGA_TT
TGA__TT
TA_TG
GA_AA
Promoter strength pos 96
TTG_TT
GA_AA
TAG_T
Promoter strength pos 104
Promoter strength pos 94
A__TG
G__GCA
CC__AGG
FE window 56
AGA_CA
GAT__CT
ATC__AA
CTC__AT
GA__GA
Promoter strength pos 137
Promoter strength pos 109
FE window 23
1216043024384.0
1088178618368.0
1034482286592.0
1021630545920.0
1003469406208.0
992876691456.0
956050702336.0
944373432320.0
925660938240.0
881984405504.0
868214636544.0
820878245888.0
792746721280.0
768958529536.0
767823183872.0
756417560576.0
750726152192.0
713649750016.0
686981578752.0
668912386048.0
665212420096.0
650805182464.0
649989718016.0
648133214208.0
630547546112.0
611006283776.0
610586853376.0
604087648256.0
602378731520.0
588877987840.0
0
0.2
0.4
0.6
0.8
1
1.2 X10^12
F score
C.
TGA__TT
Avg_motif_ATTGCGGCGG
G__GCA
Promoter strength pos 87
TTG_TT
Promoter strength pos 96
Promoter strength pos 92
AGA_TT
Promoter strength pos 159
A__TG
TGT_A
AT_TAG
TT__CA
A_T
Avg motif R_AAAATTGTGATTCATATCACATTTT
FE window 49
Promoter strength pos 137
FE window 37
A__TCC
AC__A
Avg motif R_TACCCATAT
AT_CA
AAC_AC
Promoter strength pos109
CG__A
GA__TTT
T__ATT
Avg motif CATTGCCGCA
CC__TT
Promoter strength pos 140
GA__GA
Promoter strength pos 137
Promoter strength pos 109
FE window 23
−30000
−20000
−10000
0
10000
SHAP values
High
Feature value
Low
D.
TGA__TT
Avg_motif_ATTGCGGCGG
G__GCA
Promoter strength pos 87
TTG_TT
Promoter strength pos 96
Promoter strength pos 92
AGA_TT
Promoter strength pos 159
A__TG
TGT_A
AT_TAG
TT__CA
A_T
Avg motif R_AAAATTGTGATTCATATCACATTTT
FE window 49
Promoter strength pos 137
FE window 37
A__TCC
AC__A
Avg motif R_TACCCATAT
AT_CA
AAC_AC
Promoter strength pos109
CG__A
GA__TTT
T__ATT
Avg motif CATTGCCGCA
CC__TT
Promoter strength pos 140
GA__GA
Promoter strength pos 137
Promoter strength pos 109
FE window 23
0
2000
4000
6000
8000
Average SHAP values

Figure S8. *Most influential features predictor analysis: Maximum value variable*. ***A.*** *Top 10% of frequent features from all cross-validation sets.* ***B.*** *Top 30 features F score.* ***C.*** *Top 30 features SHAP values and direction.* ***D.*** *Top 30 features averaged SHAP values.*

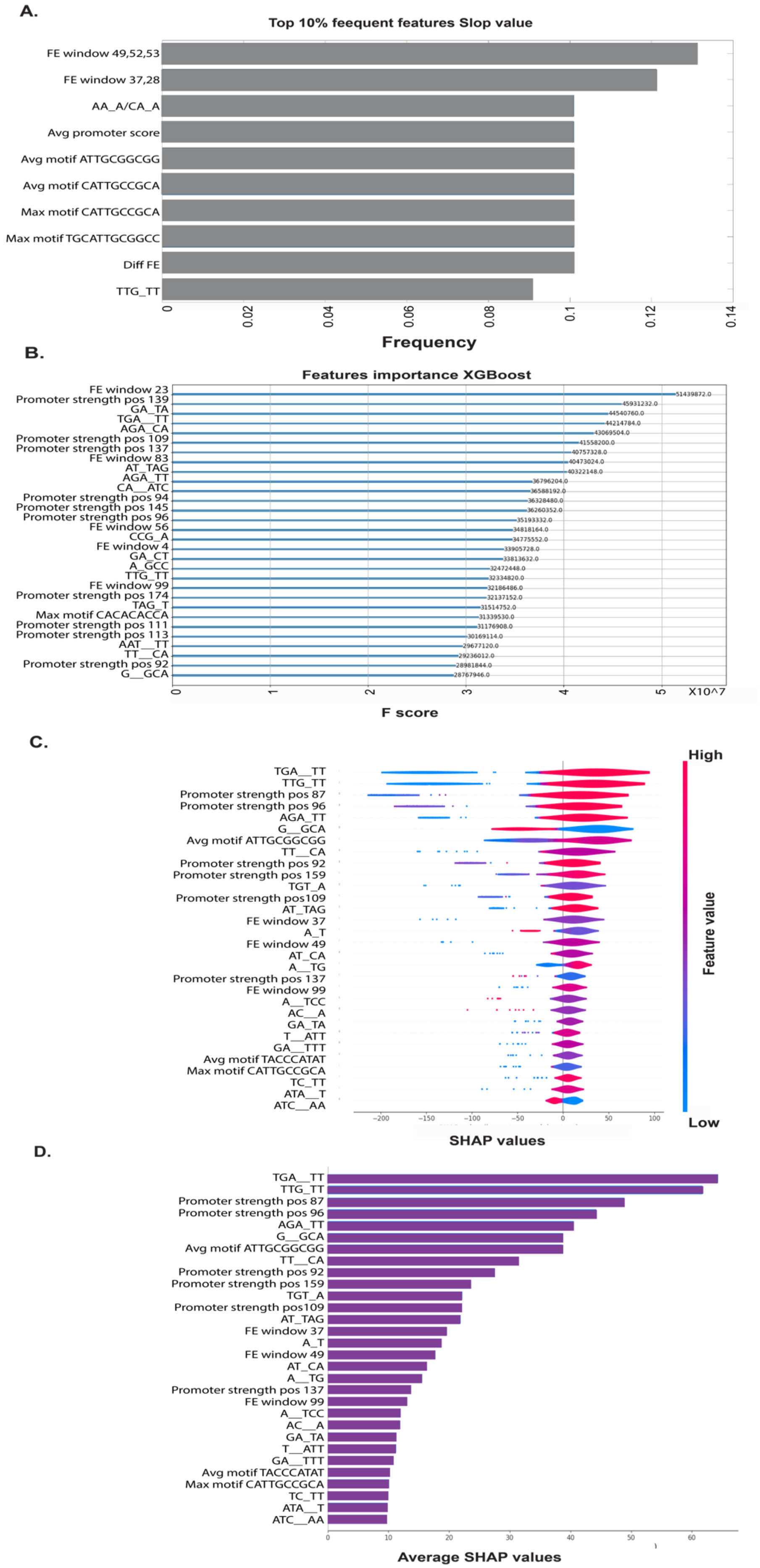

A.
Top 10% feequent features Slop value
FE window 49,52,53
FE window 37,28
AA_A/CA_A
Avg promoter score
Avg motif ATTGCGGCGG
Avg motif CATTGCCGCA
Max motif CATTGCCGCA
Max motif TGCATTGCGGCC
Diff FE
TTG_TT
0
0.02
0.04
0.06
0.08
0.1
0.12
0.14
Frequency
B.
Features importance XGBoost
FE window 23
Promoter strength pos 139
GA_TA
TGA__TT
AGA_CA
Promoter strength pos 109
Promoter strength pos 137
FE window 83
AT_TAG
AGA_TT
CA__ATC
Promoter strength pos 94
Promoter strength pos 145
Promoter strength pos 96
FE window 56
CCG_A
FE window 4
GA_CT
A_GCC
TTG_TT
FE window 99
Promoter strength pos 174
TAG_T
Max motif CACACACCA
Promoter strength pos 111
Promoter strength pos 113
AAT__TT
TT__CA
Promoter strength pos 92
G__GCA
0
1
2
3
4
5
X10^7
F score
C.
High
TGA__TT
TTG_TT
Promoter strength pos 87
Promoter strength pos 96
AGA_TT
G__GCA
Avg motif ATTGCGGCGG
TT__CA
Promoter strength pos 92
Promoter strength pos 159
TGT_A
Promoter strength pos109
AT_TAG
FE window 37
A_T
FE window 49
AT_CA
A__TG
Promoter strength pos 137
FE window 99
A__TCC
AC__A
GA_TA
T__ATT
GA__TTT
Avg motif TACCCATAT
Max motif CATTGCCGCA
TC_TT
ATA__T
ATC__AA
−200
−150
−100
−50
0
50
100
SHAP values
Feature value
Low
D.
TGA__TT
TTG_TT
Promoter strength pos 87
Promoter strength pos 96
AGA_TT
G__GCA
Avg motif ATTGCGGCGG
TT__CA
Promoter strength pos 92
Promoter strength pos 159
TGT_A
Promoter strength pos109
AT_TAG
FE window 37
A_T
FE window 49
AT_CA
A__TG
Promoter strength pos 137
FE window 99
A__TCC
AC__A
GA_TA
T__ATT
GA__TTT
Avg motif TACCCATAT
Max motif CATTGCCGCA
TC_TT
ATA__T
ATC__AA
0
10
20
30
40
50
60
Average SHAP values

Figure S9. *Most influential features predictor analysis, Slop value variable*. ***A.*** *Top 10% of frequent features from all cross-validation sets.* ***B.*** *Top 30 features F score.* ***C.*** *Top 30 features SHAP values and direction.* ***D.*** *Top 30 features averaged SHAP values.*

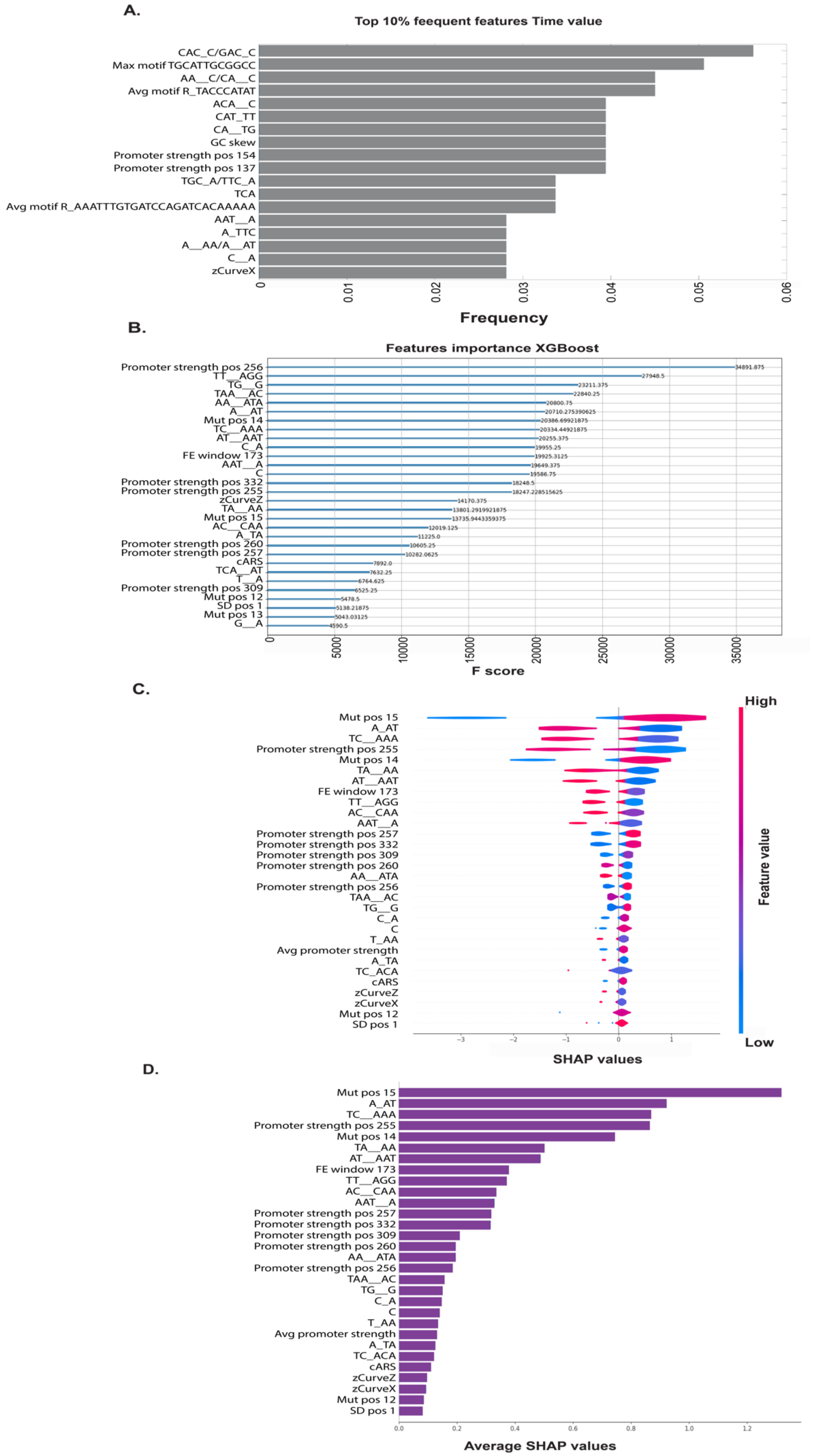
A.
Top 10% feequent features Time value
CAC_C/GAC_C
Max motif TGCATTGCGGCC
AA__C/CA__C
Avg motif R_TACCCATAT
ACA__C
CAT_TT
CA__TG
GC skew
Promoter strength pos 154
Promoter strength pos 137
TGC_A/TTC_A
TCA
Avg motif R_AAATTTGTGATCCAGATCACAAAAA
AAT__A
A_TTC
A__AA/A__AT
C__A
zCurveX
0
0.01
0.02
0.03
0.04
0.05
0.06
Frequency
B.
Features importance XGBoost
Promoter strength pos 256
TT__AGG
TG__G
TAA__AC
AA__ATA
A__AT
Mut pos 14
TC__AAA
AT__AAT
C_A
FE window 173
AAT__A
C
Promoter strength pos 332
Promoter strength pos 255
zCurveZ
TA__AA
Mut pos 15
AC__CAA
A_TA
Promoter strength pos 260
Promoter strength pos 257
cARS
TCA__AT
T__A
Promoter strength pos 309
Mut pos 12
SD pos 1
Mut pos 13
G__A
34891.875
27948.5
23211.375
22840.25
20800.75
20710.275390625
20386.69921875
20334.44921875
20255.375
19955.25
19925.3125
19649.375
19586.75
18248.5
18247.228515625
14170.375
13801.2919921875
13735.9443359375
12019.125
11225.0
10605.25
10282.0625
7892.0
7632.25
6764.625
6525.25
5478.5
5138.21875
5043.03125
4590.5
0
5000
10000
15000
20000
25000
30000
35000
F score
C.
Mut pos 15
A_AT
TC__AAA
Promoter strength pos 255
Mut pos 14
TA__AA
AT__AAT
FE window 173
TT__AGG
AC__CAA
AAT__A
Promoter strength pos 257
Promoter strength pos 332
Promoter strength pos 309
Promoter strength pos 260
AA__ATA
Promoter strength pos 256
TAA__AC
TG__G
C_A
C
T_AA
Avg promoter strength
A_TA
TC_ACA
cARS
zCurveZ
zCurveX
Mut pos 12
SD pos 1
−3
−2
−1
0
1
SHAP values
High
Feature value
Low
D.
Mut pos 15
A_AT
TC__AAA
Promoter strength pos 255
Mut pos 14
TA__AA
AT__AAT
FE window 173
TT__AGG
AC__CAA
AAT__A
Promoter strength pos 257
Promoter strength pos 332
Promoter strength pos 309
Promoter strength pos 260
AA__ATA
Promoter strength pos 256
TAA__AC
TG__G
C_A
C
T_AA
Avg promoter strength
A_TA
TC_ACA
cARS
zCurveZ
zCurveX
Mut pos 12
SD pos 1
0.0
0.2
0.4
0.6
0.8
1.0
1.2
Average SHAP values

Figure S10. *Most influential features predictor analysis: Time to max value variable*. ***A.*** *Top 10% of frequent features from all cross-validation sets.* ***B.*** *Top 30 features F score.* ***C.*** *Top 30 features SHAP values and direction.* ***D.*** *Top 30 features averaged SHAP values.*

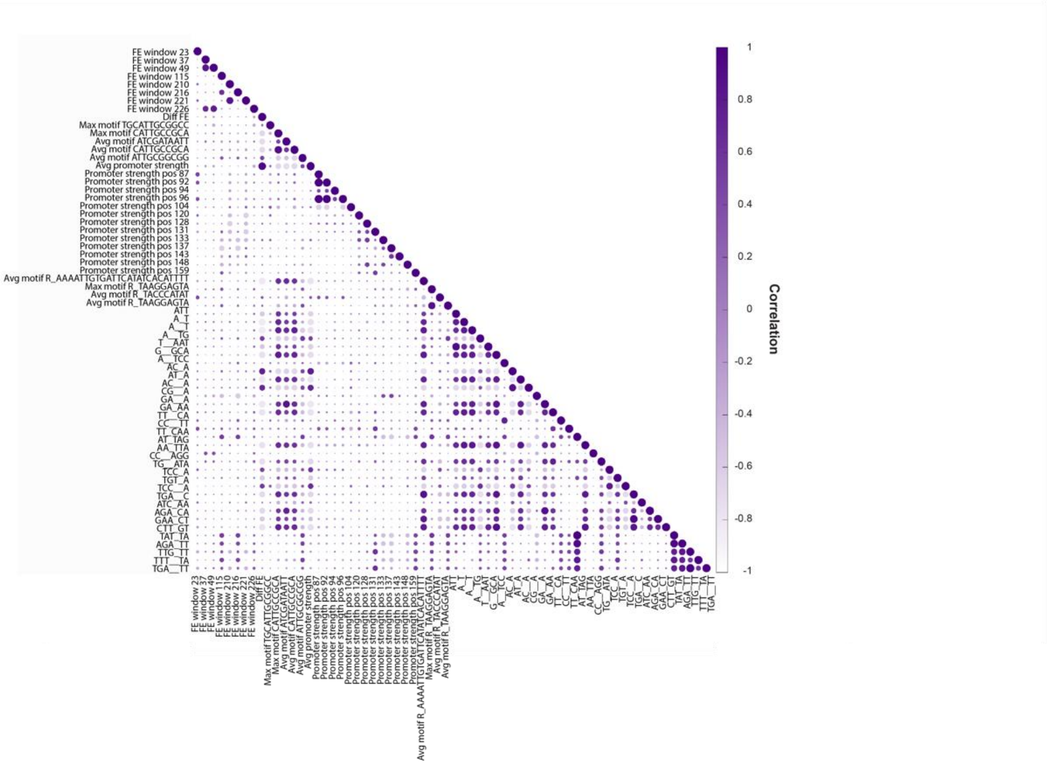

Figure S11. *The Spearman correlation between each pair of the most important features was calculated, and the correlation among the most important features is depicted, with the color and size of the circles indicating the strength of the correlation.*

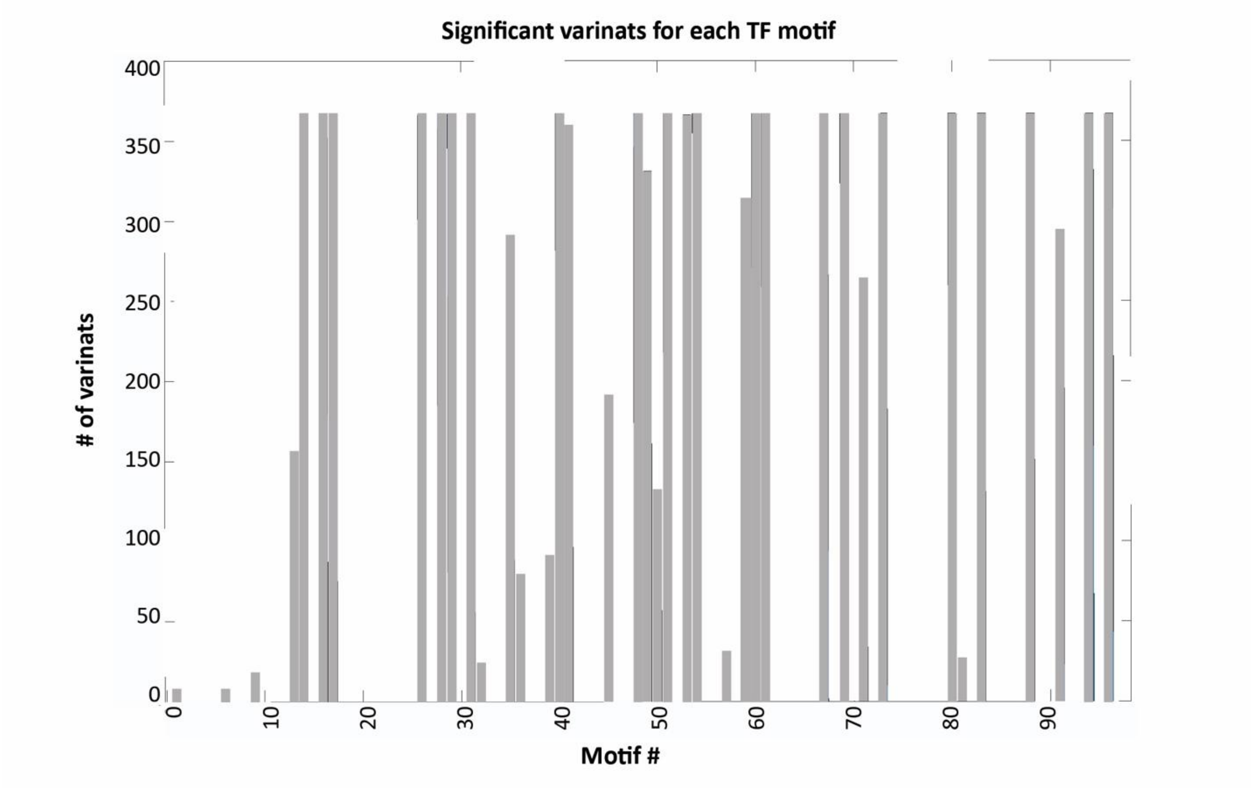

Figure S12. Significant TF motifs. For each motif, the number of variants whose PSSM scores exceeded the significance threshold indicates motif-specific enrichment patterns across the dataset. Of note, all variants were significant for 20 motifs.
